# An Anisotropic Gold‐Palladium Heterostructured Nanosystem for Synergistically Overcoming Radioresistance and Enhancing Melanoma Radioimmunotherapy

**DOI:** 10.1002/advs.202500492

**Published:** 2025-06-17

**Authors:** Cheng Chen, Yuqi Huang, Wandong Wang, Minghao Chao, Weiguo Sun, Yinghui Kong, Guan Jiang, Yong Gao, Fenglei Gao

**Affiliations:** ^1^ Department of Dermatology The Affiliated Huaian No. 1 People's Hospital of Nanjing Medical University Jiangsu 223300 P. R. China; ^2^ Department of Dermatology The Affiliated Suzhou Hospital of Nanjing Medical University Jiangsu 215000 P. R. China; ^3^ Jiangsu Key Laboratory of New Drug Research and Clinical Pharmacy Xuzhou Medical University Jiangsu 221004 P. R. China; ^4^ Department of Dermatology University Medical Center Groningen University of Groningen Groningen 9713 GZ the Netherlands; ^5^ Department of Dermatology Affiliated Hospital of Xuzhou Medical University Xuzhou Jiangsu 221002 P. R. China; ^6^ Department of Oncology The Affiliated Huaian No. 1 People's Hospital of Nanjing Medical University Jiangsu 223300 P. R. China

**Keywords:** melanoma, mild hyperthermia therapy, nanovaccine, radioimmunotherapy

## Abstract

Radiotherapy (RT) has recently reemerged as a promising approach for melanoma treatment because of its potential to trigger abscopal effects. However, the intrinsic radioresistance of melanoma significantly diminishes RT‐induced DNA damage and the subsequent release of immunostimulatory molecules, thereby impairing systemic antitumor immunity. To overcome these challenges, a multifunctional anisotropic Au‐Pd heterostructured nanosystem (APSMR) is developed that incorporates a plasmonically enhanced Au‐Pd core, with a shell composed of a biodegradable, Mn‐doped targeting peptide. The nanosystem integrates photothermal, radiotherapeutic, and immunomodulatory functions. Under 1064 nm laser irradiation, APSMR generates reactive oxygen species (ROS) via plasmon‐driven catalysis and Mn‐mediated Fenton‐like reactions. Concurrently, mild hyperthermia (HT) promotes oxygenation and disrupts DNA repair pathways, resulting in multi‐directional DNA damage and an increase in immunogenic cell death (ICD). Furthermore, the release of Mn^2^⁺ ions activates the cGAS–STING pathway, which synergizes with ICD to promote systemic antitumor immunity. Notably, APSMR treatment also upregulates PD‐L1 expression, thereby sensitizing tumors to immune checkpoint blockade. Collectively, APSMR offers a potent and synergistic strategy to amplify RT‐driven tumor vaccination and improve therapeutic responses against metastatic melanoma.

## Graphical abstract

A multifunctional anisotropic Au‐Pd heterostructured nanosystem is developed that incorporates a plasmonically enhanced Au‐Pd core, with a shell composed of a biodegradable, Mn‐doped targeting peptide. The nanosystem integrates photothermal, radiotherapeutic, and immunomodulatory functions.

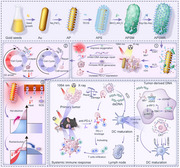

## 1 Introduction

Tumor vaccines are a crucial component of cancer immunotherapy, aimed at converting tumors into platforms for presenting tumor‐associated antigens (TAAs), thereby eliciting robust and diverse antitumor immune responses.^[^ ^1^, ^2^, ^3^, ^4^ ^]^ Alongside the rapid progress of immunotherapy, radiotherapy (RT) has regained attention due to its abscopal effects.^[^ ^5^, ^6^, ^7^ ^]^ Researchers have begun evaluating the potential value of RT as an in situ tumor vaccine.^[^ ^8^ ^]^ However, melanoma typically exhibits poor radiation absorption and intrinsic radioresistance, thereby diminishing RT‑induced DNA damage^[^ ^9^, ^10^, ^11^ ^]^ and limiting the generation of TAAs and immunostimulatory molecules within the tumor microenvironment. This significantly restricts systemic antitumor immunity; indeed, only 46 cases of RT‐induced abscopal effects were documented between 1969 and 2014,^[^ ^12^ ^]^ emphasizing the need for enhanced strategies to improve radioimmunotherapy, particularly in highly aggressive melanoma.

Enhancing RT‐induced immunogenic cell death (ICD) has represented a promising strategy to overcome these challenges.^[^ ^13^, ^14^ ^]^ During the ICD process, tumor cells expose calreticulin (CRT) on their surfaces and release adenosine triphosphate (ATP) and high mobility group box 1 (HMGB1), thereby providing critical antigens and immune adjuvants necessary for effective tumor vaccination. Although high‐dose RT facilitates antigen release, it also increases adverse effects and induces DNA exonuclease Trex1, ultimately reducing overall tumor immunogenicity.^[^ ^15^, ^16^ ^]^ To address these issues, low‐dose radiation sensitizers that incorporate high atomic number (Z) elements (e.g., Hf^[^ ^17^ ^]^ and Au^[^ ^18^ ^]^) have been extensively investigated. These sensitizers enhance X‐ray energy deposition and reactive oxygen species (ROS) generation,^[^ ^19^ ^]^ thereby enhancing RT‐induced ICD.^[^ ^20^ ^]^ However, due to melanoma's multifaceted resistance mechanisms,^[^ ^21^, ^22^, ^23^, ^24^ ^]^ these strategies alone yield only modest immune enhancements. Recent research further suggests that enhancing RT‑induced activation of the cGAS–STING pathway may provide an additional avenue to boost immune responses.^[^ ^25^, ^26^ ^]^ This mechanism partly explains why simple radiation sensitization strategies have not significantly improved RT‐induced in situ vaccination responses. Melanoma radioresistance is characterized by insufficient DNA double‐strand breaks. Moreover, tumor cells employ repair mechanisms, including non‐homologous end joining (NHEJ) and homologous recombination (HR), to reduce cGAS‐STING pathway activation, resulting in inadequate type I interferon secretion. With the introduction of the “metal immunology” concept,^[^ ^27^, ^28^ ^]^ researchers have developed various manganese (Mn)‐based nanodrugs designed to activate the STING pathway.^[^ ^29^, ^30^, ^31^ ^]^ However, these Mn‐based nanodrugs still encounter challenges, including acute toxicity, aggregation, and rapid metabolism, all of which limit their effectiveness in modulating tumor immune responses.

Mild hyperthermia therapy (HT), which involves heating tumors to below 43 °C for about one hour, has emerged as a potent radiosensitizer.^[^ ^32^, ^33^ ^]^ Early studies primarily attributed the sensitizing effects of HT to direct thermal damage and reduced tumor hypoxia.^[^ ^33^ ^]^ However, more recent investigations have revealed that HT also disrupts key proteins involved in the NHEJ and HR repair pathways.^[^ ^34^ ^]^ Building on this understanding, combining HT with RT holds promise for significantly enhancing DNA damage, inducing ICD, and activating the cGAS‐STING pathway. Such a combination offers a novel and synergistic approach to further amplify immune responses following RT. In this regard, gold nanorods (Au NRs) have become ideal candidates for photothermal therapy (PTT) owing to their efficient synthesis, chemical stability, and robust near‐infrared (NIR) light absorption.^[^ ^35^, ^36^, ^37^ ^]^ Despite these advantages, optimizing their catalytic and photothermal properties remains a challenge. To address this limitation, researchers have explored the hybridization of Au NRs with palladium (Pd), resulting in Au‐Pd heterostructures that significantly enhance both catalytic and photothermal efficiencies.^[^ ^38^, ^39^ ^]^ This modification leads to the generation of abundant hot electrons, which, in turn, further enhance ROS production, thereby improving therapeutic efficacy.^[^ ^40^ ^]^ Furthermore, balancing safety and functionality is critical for clinical translation of Mn‐based inorganic nanomaterials. To address this, an exemplary case is hollow mesoporous manganese silicate (MnSiO₃), where the Mn‐O bond exhibits heightened sensitivity to the acidic and reductive conditions of the tumor microenvironment. This sensitivity triggers “manganese extraction,” which degrades the nanostructure and releases Mn^2^⁺.^[^ ^41^ ^]^ The release of Mn^2^⁺ then initiates a Fenton‐like reaction, generating ROS that collaboratively enhance the effectiveness of RT. Notably, this mechanism not only mitigates potential biosafety issues associated with nanomaterials but also enriches their therapeutic capabilities, providing strong support for the safe and effective application of manganese‐based inorganic nanomaterials in clinical settings.

**Scheme 1:**
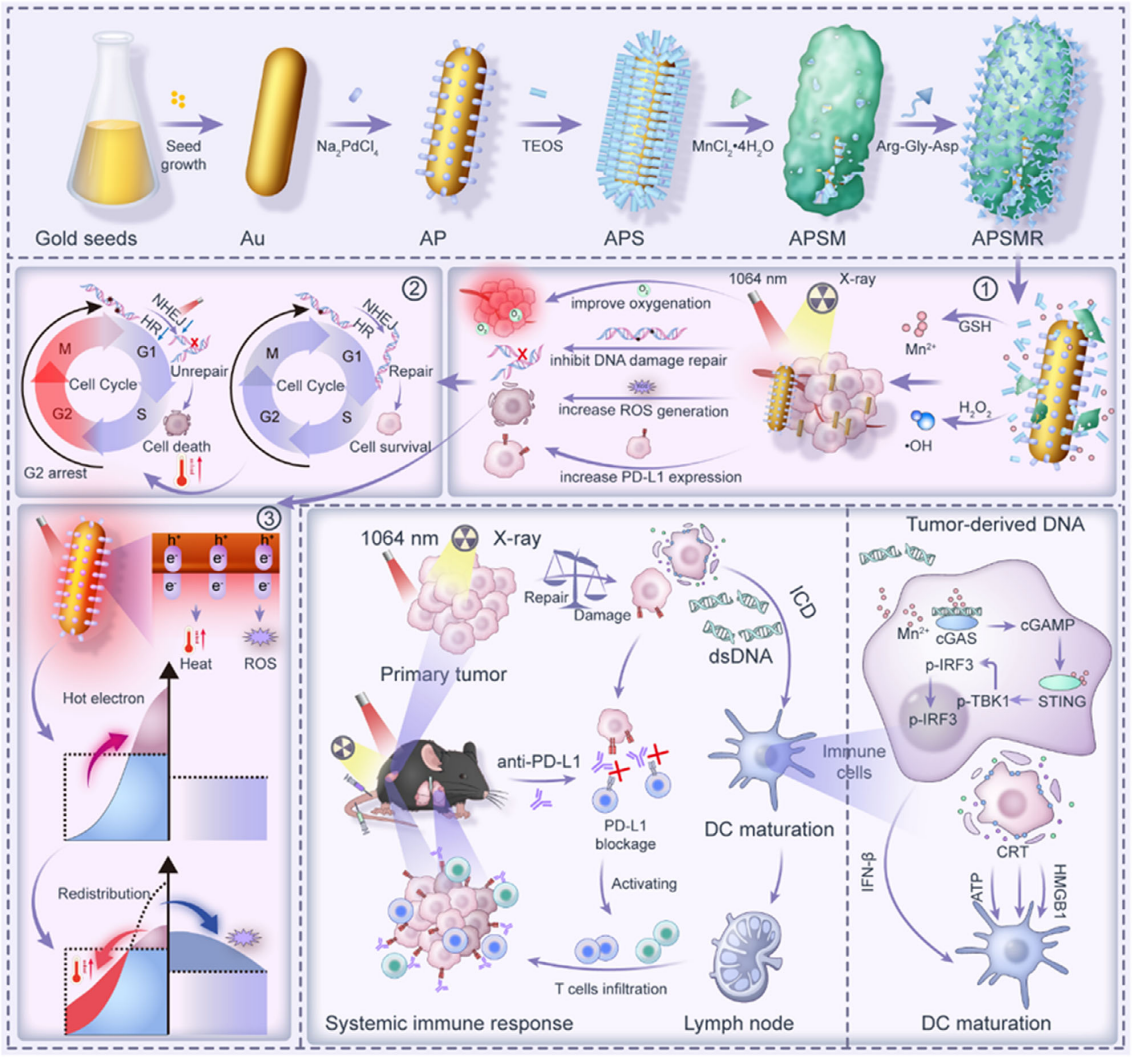
Schematic diagram illustrating the synthesis process of APSMR and its underlying therapeutic mechanism for melanoma.

Based on these considerations, we designed an anisotropic Au‐Pd heterostructured nanosystem (APSMR) to facilitate integrated radioimmunotherapy. Its core features a gold‐palladium (Au‐Pd) nanoparticle heterostructure with a “super antenna” configuration, while its shell consists of a biodegradable, Mn‐doped targeting peptide (Arg‐Gly‐Asp).^[^ ^42^, ^43^ ^]^ As illustrated in **Scheme** **1**, APSMR exploits combined photothermal, radiotherapeutic, and immunological mechanisms to stimulate robust tumor vaccination. The targeting peptide enhances selective nanoparticle binding to melanoma cells, while Mn‐shell degradation under acidic and reductive conditions initiates a Fenton‐like reaction, generating hydroxyl radicals (•OH) and weakening melanoma antioxidant defenses. Upon 1064 nm laser irradiation,^[^ ^44^, ^45^ ^]^ the APSMR's heterostructure exhibits plasmon‐driven catalytic activity, converting hot electrons into additional ROS. Meanwhile, HT promotes tumor oxygenation and disrupts DNA repair processes, thereby amplifying DNA damage and ICD. The release of Mn^2^⁺ further potentiates activation of the cGAS–STING signaling pathway, significantly amplifying systemic immune responses. Another critical point to consider is that APSMR‐mediated HT combined with RT may elevate PD‐L1 expression in melanoma,^[^ ^46^, ^47^ ^]^ thereby boosting the efficacy of anti–PD‐L1 immune checkpoint therapy and overcoming the limitations of single therapeutic strategies. In conclusion, the APSMR nanosystem synergistically integrates RT and HT providing potent local tumor control and amplifying systemic immune responses by enhancing RT‐induced vaccine responses and combining treatment with anti‐PD‐L1 immune checkpoint inhibitors. This integrated therapeutic strategy holds significant promise for effectively suppressing distant melanoma metastases.

## 2 Results and Discussion

### 2.1 Mild Hyperthermia Inhibits DNA Damage Repair

**Figure 1:**
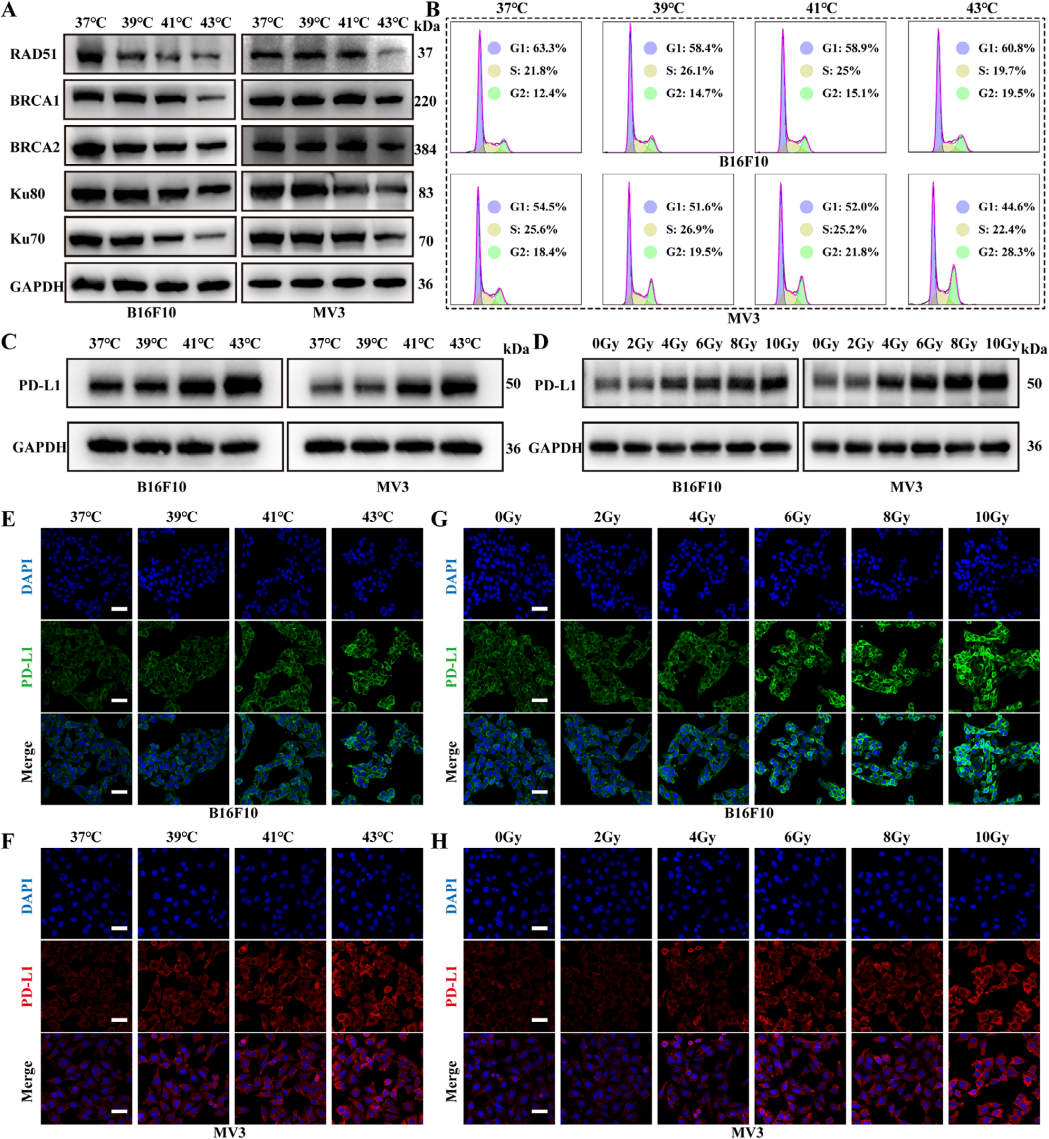
Effects of HT on DNA damage repair and its combined impact with RT on PD‐L1 expression in vitro. A) Western blot analysis of key proteins in the NHEJ and HR pathways across various temperatures. B) Cell cycle flow cytometry profiles of B16F10 and MV3 cells across various temperatures. C) PD‐L1 protein expression in B16F10 and MV3 cells across a range of temperatures. D) PD‐L1 protein expression in B16F10 and MV3 cells after varying radiation doses. E,F) Immunofluorescence images showing PD‐L1 expression in B16F10 and MV3 cells at various temperatures (scale bar: 50 µm). G,H) Immunofluorescence images displaying PD‐L1 expression in B16F10 and MV3 cells after various radiation doses (scale bar: 50 µm).

The capacity of HT to enhance tumor oxygenation has been well documented. However, an additional and potentially pivotal mechanism by which HT enhances radiosensitivity may lie in the modulation of radiation‐induced DNA damage repair. These lesions are primarily repaired through two canonical pathways: NHEJ and HR. To investigate this possibility, we analyzed the expression of key proteins involved in these pathways, including Rad51, Ku70, Ku80, BRCA1, and BRCA2 (**Figure** **1**A). The results showed a pronounced reduction in the expression of these proteins in MV3 and B16F10 cells following exposure to 43 °C for 1 h, indicating that HT at 43 °C suppresses DNA double‐strand break repair. Given the tight coordination between DNA damage repair and cell cycle dynamics, we next analyzed cell cycle progression via flow cytometry (Figure 1B). Compared to other temperature conditions, HT at 43 °C led to an increased accumulation of cells in the G2 phase, the most radiosensitive phase of the cell cycle. Therefore, HT at 43 °C demonstrated substantial potential for enhancing radiosensitivity in melanoma.

### 2.2 HT and RT Increase PD‐L1 Expression in Melanoma

The mechanisms underlying the immunosuppressive or immunostimulatory effects of RT are highly complex. Nevertheless, a deeper understanding of the immune system's role in RT could unveil novel therapeutic opportunities for melanoma management. Beyond the previously noted “abscopal effect,” certain studies have indicated that elevated temperature and X‐ray exposure may modulate PD‐L1 expression in tumor cells. Although this phenomenon has yet to be fully validated in melanoma, we conducted preliminary experiments using two commonly employed melanoma cell lines, B16F10 and MV3, to explore this regulatory effect. Our results indicated that, compared to other treatment conditions, the 43 °C group displayed higher PD‐L1 expression (Figure 1C). Moreover, exposure to 4 Gy or higher radiation doses markedly elevated PD‐L1 expression in both B16F10 and MV3 cells (Figure 1D). These findings were further validated by immunofluorescence assays (Figure 1E–H). Notably, PD‐L1 expression was further elevated when HT was combined with RT, as illustrated in Figure S1 (Supporting Information). Overall, these results provided a solid rationale for combining RT and hyperthermia with anti–PD‐1/PD‐L1 therapy, offering valuable insights for predicting treatment outcomes and identifying patients most likely to benefit from such combination strategies.

### 2.3 Synthesis and Characterization of APSMR

**Figure 2:**
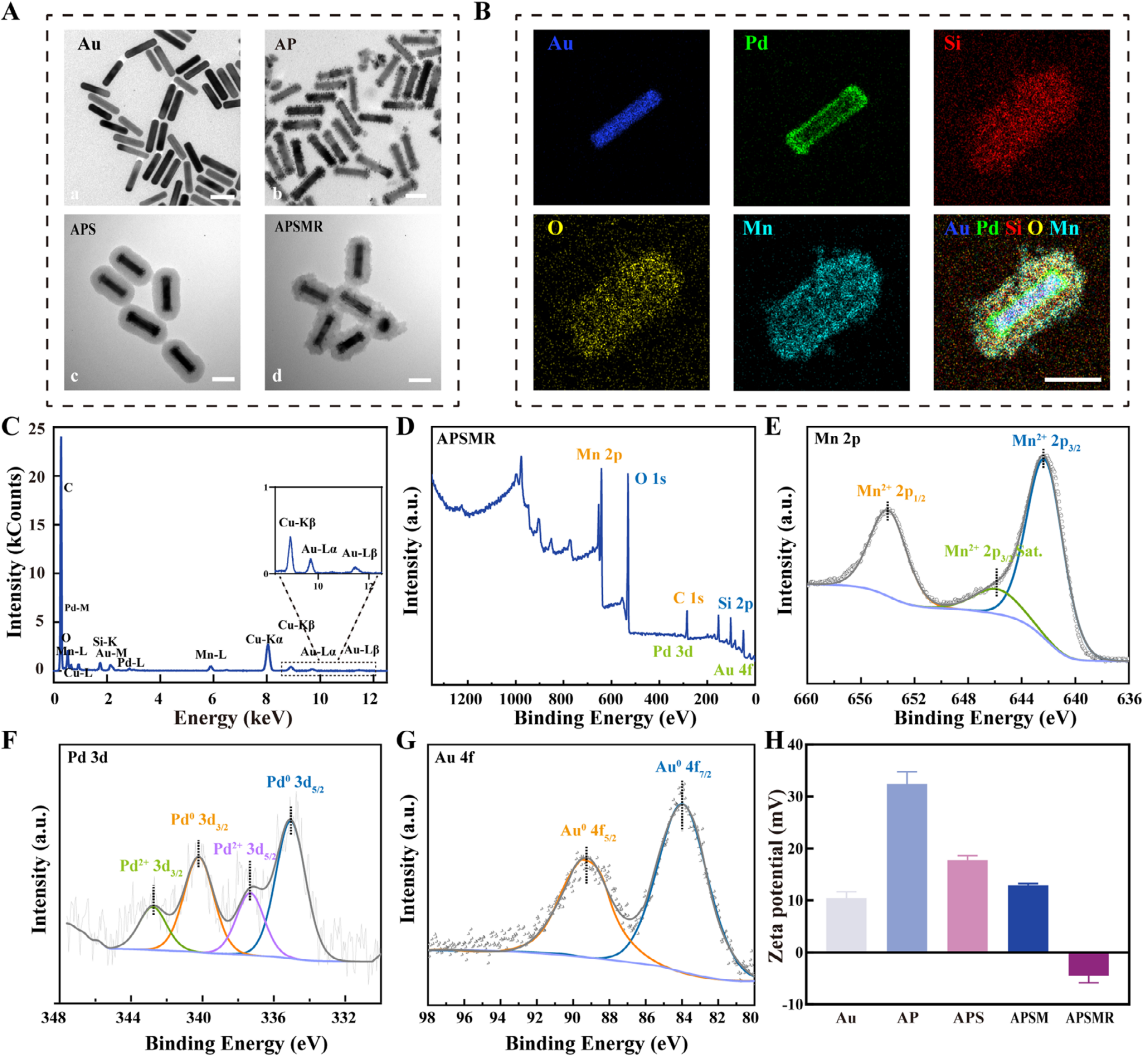
Preparation and characterization of APSMR. A) TEM images of nanoparticles at each synthesis step (scale bar: 50 nm). B) Elemental mapping of APSMR (scale bar: 50 nm). C) EDS spectrum of APSMR. D) High‐resolution full XPS spectrum of APSMR. E) High‐resolution XPS Mn 2p spectrum of APSMR. F) High‐resolution XPS Pd 3d spectrum of APSMR. G) High‐resolution XPS Au 4f spectrum of APSMR. H) Zeta potential of nanoparticles at each synthesis step.

In this study, we successfully synthesized monodisperse and high‐yield Au NRs by optimizing reactant ratios. As shown in **Figure** **2**A‐a, the synthesized Au NRs have a size below 100 nm with an aspect ratio of ≈4, offering optimal conditions for strong NIR II absorption in the APSMR system. Subsequently, Pd nanoparticles were deposited onto the Au NRs surface via a redox reaction with Na_2_PdCl_4_. Pd nanoparticles were uniformly arranged on the Au NRs surface, forming an Au‐Pd nanostructure (AP) resembling an antenna (Figure 2A‐b). Next, the core‐shell structure of Au‐Pd‐SiO_2_ (APS) was synthesized on the Au‐Pd surface by a hydrothermal method. Figure 2A‐c illustrated that the presence of mesoporous silica on the particle surface significantly increased the available reactive surface area for subsequent Mn doping. Under alkaline conditions and elevated temperatures, a reaction with MnCl₂·4H₂O yielded Au‐Pd‐MnSiO₃ (APSM) nanoparticles. Finally, covalent modification with RGD peptides, which specifically target melanoma cells, yielded the APSMR nanoparticles (Figure 2A‐d). Compared with the undoped nanoparticles, APSMR exhibited a noticeably rougher surface morphology, indirectly confirming successful peptide functionalization (Figure S2, Supporting Information). The APSMR nanoparticles were further characterized to confirm their composition and structure. As shown in Figures 2B and S3 (Supporting Information), the EDS detected the presence of Au, Pd, Si, Mn, and O elements. Specifically, Pd was uniformly distributed over the Au surface, confirming the successful formation of the Au‐Pd antenna‐like heterostructure. Moreover, Si, Mn, and O elements were uniformly distributed in the nanoparticle shell, indicating the successful construction of APSMR nanoparticles. Additionally, the EDS spectra (Figure 2C) also exhibited characteristic peaks corresponding to Au, Pd, Si, Mn, and O. A compositional table (Figure S4, Supporting Information) detailed the atomic and mass ratios, further validating the synthesis. XPS spectra provided additional chemical state confirmation. The full survey spectrum (Figure 2D) revealed the presence of Mn, Si, O, C, Pd, and Au. In the Mn 2p spectrum (Figure 2E), two peaks at 642.25 and 653.95 eV correspond to the 2p₃/₂ and 2p₁/₂ orbitals of Mn^2^⁺, indicating that Mn existed predominantly in the divalent oxidation state. The Pd 3d spectrum (Figure 2F) displayed peaks at 335.06 and 340.21 eV, corresponding to Pd⁰ 3d₅/₂ and 3d₃/₂ orbitals, confirming its metallic state. Similarly, Au peaks were observed at 83.95 and 89.29 eV in the Au 4f spectrum (Figure 2G), indicating that Au also resided mainly in its metallic form (Au⁰). As shown in Figure 2H, surface zeta potential measurements further confirmed successful RGD modification: the potential decreased from +12.88 mV (APSM) to −4.6 mV (APSMR), consistent with successful peptide conjugation.

### 2.4 Physicochemical Properties of APSMR

**Figure 3:**
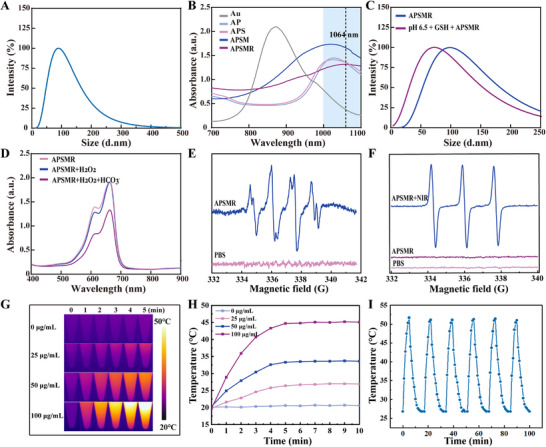
Characterization and physicochemical properties. A) Hydrodynamic diameter distribution of APSMR nanoparticles. B) UV‐Vis‐NIR absorption spectra of APSMR nanoparticles at various synthesis steps (100 µg mL^−1^). C) Hydrodynamic diameter distribution of APSMR nanoparticles pre‐and post‐degradation. D) UV‐Vis‐NIR absorption spectra of MB degradation. E) ESR spectra of •OH generated by degraded APSMR nanoparticles (1064 nm, 0.5 W cm^−^^2^). F) ESR spectra of ^1^O_2_ generated by APSMR nanoparticles (1064 nm, 0.5 W cm^−^^2^). G) Near‐infrared thermal imaging of APSMR aqueous solutions at concentrations of 0, 25, 50, and 100 µg mL^−1^ (0.5 W cm^−^^2^, 5 min). H) Corresponding photothermal temperature curves of APSMR aqueous solutions (0.5 W cm^−^^2^, 10 min). I) Photothermal stability curve of APSMR nanoparticles across six heating‐cooling cycles (1064 nm, 0.5 W cm^−^^2^).

The APSMR nanoparticles underwent comprehensive physicochemical characterization after successful fabrication. DLS analysis showed a narrow size distribution with a predominant peak ≈100 nm (**Figure** **3**A). We then examined the absorbance spectra to determine the optical absorption peaks. As shown in Figure 3B, the Au nanorods had an absorption peak at ≈870 nm. Notably, the vertically arranged Pd nanoparticles on the Au NR surface induced a redshift of the absorption peak to ≈1030 nm, thus placing it within the NIR II region (>1000 nm). In contrast, neither the APS nor the APSM nanoparticles demonstrated significant shifts in their absorption peaks relative to the Au‐Pd nanoparticles. However, the modified APSMR nanoparticles exhibited a further shift to 1060 nm, suggesting improved optical properties beneficial for deeper tissue penetration under 1064 nm laser irradiation. Furthermore, the metal‐doped APSMR nanoparticles were engineered to degrade in weakly acidic or reductive microenvironments. The particle size distribution of the simulated degradation group shifted to smaller sizes compared with the non‐degraded group, indicating nanoparticle degradation (Figure 3C). This finding was corroborated by TEM images of the degraded nanoparticles (Figure S5, Supporting Information). Additionally, to assess whether Mn^2^⁺ ions released during degradation could catalyze a Fenton‐like reaction, MB was used as an indicator.

The absorption peak of MB significantly diminished in the presence of Mn^2^⁺ and H₂O₂, confirming the occurrence of a Fenton‐like reaction (Figure 3D). These results suggest that Mn^2^⁺ released from the degraded APSMR nanoparticles can effectively generate •OH and counteract the antioxidative effects of the tumor microenvironment on ROS. The nanoparticles maintained consistent sizes in saline, PBS, and culture medium, confirming their stability (Figure S6, Supporting Information). Figure S7 (Supporting Information) further indicated that APSMR has excellent colloidal stability in serum, suggesting significant potential for in vivo applications. To elucidate the types of ROS generated, we performed ESR analysis. As shown in Figure 3E, the addition of HCO_3_^−^ and H₂O₂ to the degraded nanoparticles produced a classic 1:2:2:1 signal, confirming •OH generation. We also examined APSMR's capacity to generate¹O₂. Under 1064 nm laser irradiation, APSMR exhibited a characteristic 1:1:1 signal, indicative of ¹O₂ production (Figure 3F). Next, we examined APSMR's photothermal properties. A 100 µg mL⁻¹ suspension was irradiated with a 1064 nm laser at 0.5 W cm^−2^ (Figure S8, Supporting Information), resulting in a marked heating effect. We subsequently acquired near‐infrared thermal images and corresponding temperature profiles at 0, 25, 50, and 100 µg mL⁻¹ (Figure 3G,H). As shown in Figure 3G, the pure water group, which did not contain nanoparticles, exhibited a negligible temperature increase under 1064 nm irradiation, rising from 20.1 °C to only 20.6 °C. By contrast, APSMR suspensions at various concentrations displayed significant temperature increases, with the 100 µg mL⁻¹ sample reaching 45.1 °C, surpassing the 43 °C threshold for mild photothermal therapy (PTT). Furthermore, Figures S9 (Supporting Information) and **3I** demonstrated that the 100 µg mL⁻¹ APSMR suspension maintained a stable temperature profile after six heating‐cooling cycles, indicating robust photothermal stability.

### 2.5 Cellular Uptake and Cytotoxicity of APSMR

**Figure 4:**
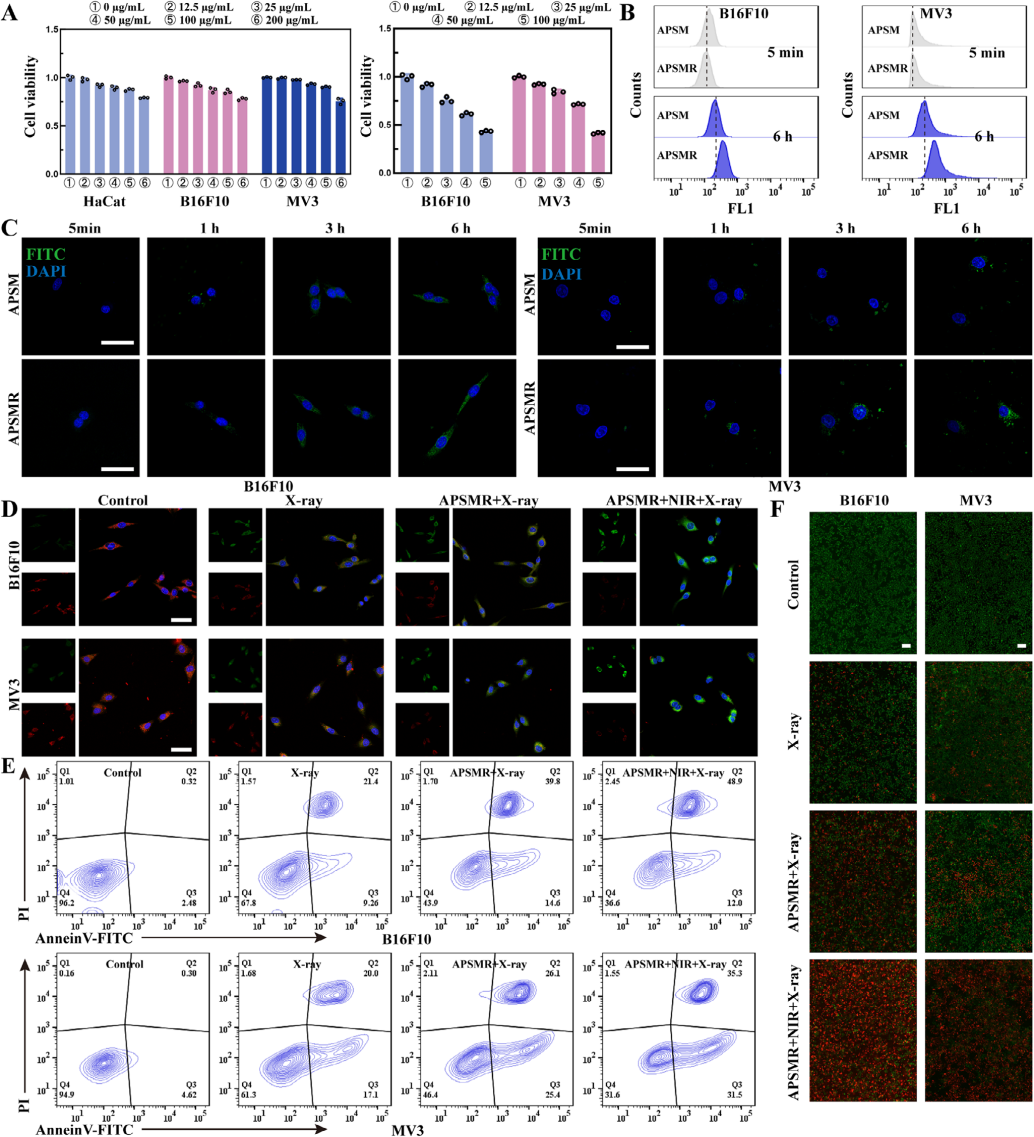
Cellular uptake and cytotoxicity of APSMR. A) CCK‐8 analysis of nanoparticle cytotoxicity at various concentrations on HaCat, B16F10, and MV3 cells. B) Flow cytometric analysis of cellular uptake of APSM and APSMR by B16F10 and MV3 cells at various time points. C) Fluorescence microscopy images of B16F10 and MV3 cells at multiple time points (5 min, 1 h, 3 h, and 6 h) post‐uptake of APSM‐FITC and APSMR‐FITC nanoparticles (scale bar: 50 µm). D) Fluorescence imaging of mitochondrial membrane potential in B16F10 and MV3 cells stained with JC‐1 dye (scale bar: 50 µm). E) Flow cytometric analysis of B16F10 and MV3 cells stained with Annexin V‐FITC/PI. F) Fluorescence microscopy images of live/dead B16F10 and MV3 cells stained with calcein‐AM/PI (scale bar: 200 µm).

As exhibited in **Figure** **4**A, at concentrations up to 200 µg mL^−1^, all groups displayed cell viability above 80%, indicating the high biocompatibility of APSMR. To identify the optimal treatment concentration, B16F10 and MV3 cells were incubated with various nanoparticle concentrations under identical treatment conditions (RT: 4 Gy; HT: 1064 nm, 0.5 W cm^−^^2^, 1 h). Compared with the untreated controls, the 100 µg mL^−1^ nanoparticles group exhibited significantly reduced cell viability (Figure 4B). Therefore, considering both biotoxicity and therapeutic efficacy, 100 µg mL^−1^ was chosen as the concentration for subsequent experiments. Because cellular uptake of APSMR plays a critical role in the therapeutic efficacy against melanoma. To assess this, B16F10 and MV3 cells were incubated with APSM‐FITC or APSMR‐FITC nanoparticles. The APSMR‐FITC group displayed more pronounced green fluorescence signals than the APSM‐FITC group (Figure 4B,C), indicating that APSMR effectively targeted melanoma cells. To further validate APSMR's targeting ability, a similar experiment was conducted using 3D melanoma spheroids formed by B16F10 and MV3 cells. Z‐axis imaging of these spheroids showed stronger green fluorescence with APSMR‐FITC than with APSM‐FITC, both at the periphery and within the spheroid (Figure S10, Supporting Information).

The generation of ROS is directly related to the damage efficiency of RT in melanoma cells. We employed the DCFH‐DA assay to detect ROS production following various treatments. Melanoma cells in the RT group produced stronger green fluorescence signals than the control group, whereas the APSMR+RT group showed even higher fluorescence intensity (Figure S11, Supporting Information). This increase was attributed to the Mn^2^⁺‐mediated Fenton‐like reaction and enhanced radiation absorption by APSMR nanoparticles. Moreover, the APSMR+RT+NIR group displayed the strongest green fluorescence, primarily due to HT's supplementary inhibition of DNA damage repair in tumor cells. Flow cytometry analysis further confirmed these findings, as depicted in Figure S12 (Supporting Information), the RT group's signal peak shifted to the right versus the control, while the APSMR+RT+NIR group showed an even greater rightward shift, indicating increased ROS production. Figure 4D suggested that ROS production induced by the treatment damaged the mitochondrial membrane potential in B16F10 and MV3 cells, as observed in JC‐1 fluorescence assays. Flow cytometry apoptosis assays (Figure 4E) indicated a progressive increase in the proportion of cells in the Q2 quadrant, indicating substantial membrane damage and resulting in both membrane and nuclear staining. Finally, live/dead staining experiments confirmed that APSMR effectively induced cell death in B16F10 and MV3 cells, with the combination therapy group showing a markedly higher proportion of dying tumor cells (Figure 4F).

### 2.6 Radiation Sensitization of APSMR In Vitro

**Figure 5:**
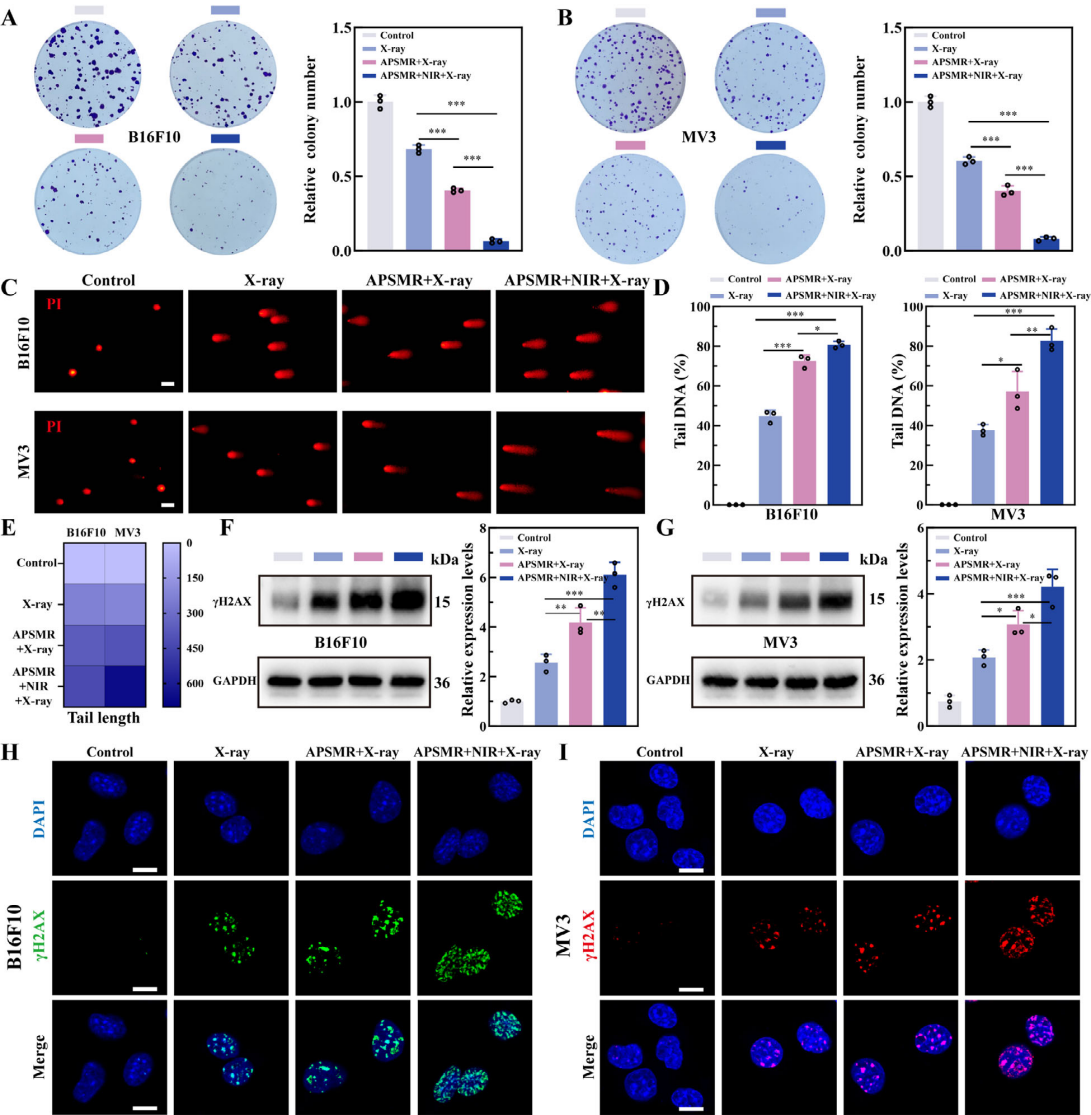
Radiation sensitization of APSMR in vitro. A,B) Representative colony formation images and corresponding statistical analysis of colony counts in B16F10 and MV3 cells following various treatments. C) Fluorescence images from comet assays in B16F10 and MV3 cells after various treatments (scale bar: 100 µm). D) Statistical analysis of DNA content in comet tails from B16F10 and MV3 cells after the comet assay. E) Heatmap of tail moment from comet assays in B16F10 and MV3 cells post‐treatment. F,G) Protein expression levels of γH2AX in B16F10 and MV3 cells following various treatments. H,I) Immunofluorescence images of γH2AX in B16F10 and MV3 cells after treatment (scale bar: 10 µm). Data are presented as mean values± SD. *p < 0.05, **p < 0.01, and ***p < 0.001.

Following preliminary cytotoxicity assessments, we further evaluated the DNA damage induced by APSMR in B16F10 and MV3 cells. As illustrated in **Figure** **5**A,B, the colony formation assay revealed that the APSMR+RT+NIR group formed the fewest colonies compared to the other groups, suggesting robust radiation‐sensitizing effects of the synthesized nanoparticles. In the comet assay (Figure 5C), no significant DNA tail migration was observed in the control group for either cell line. By contrast, DNA tail lengths gradually increased across the other three experimental groups, indicating a corresponding rise in DNA damage. CASP software analysis (Figure 5D,E) revealed a significant increase in comet tail DNA content and tail length in the APSMR+RT group versus the RT group, with an even greater rise observed in the APSMR+RT+NIR group. Phosphorylated H2AX (γH2AX) is a sensitive indicator of DNA double‐strand breaks, with its expression correlating with the severity of DNA damage. As depicted in (Figure 5F,G), γH2AX expression was minimal in untreated melanoma cells but gradually increased in the RT, APSMR+RT, and APSMR+RT+NIR groups. In agreement with these observations, γH2AX immunofluorescence results (Figure 5H,I) revealed no discernible fluorescent signal foci in the control group, while both fluorescence intensity and particle accumulation increased progressively in the RT, APSMR+RT, and APSMR+RT+NIR groups. Collectively, these results indicated that combining RT and HT induced more extensive DNA double‐strand break damage in melanoma cells.

### 2.7 APSMR Elicits Robust ICD and STING Activation

**Figure 6:**
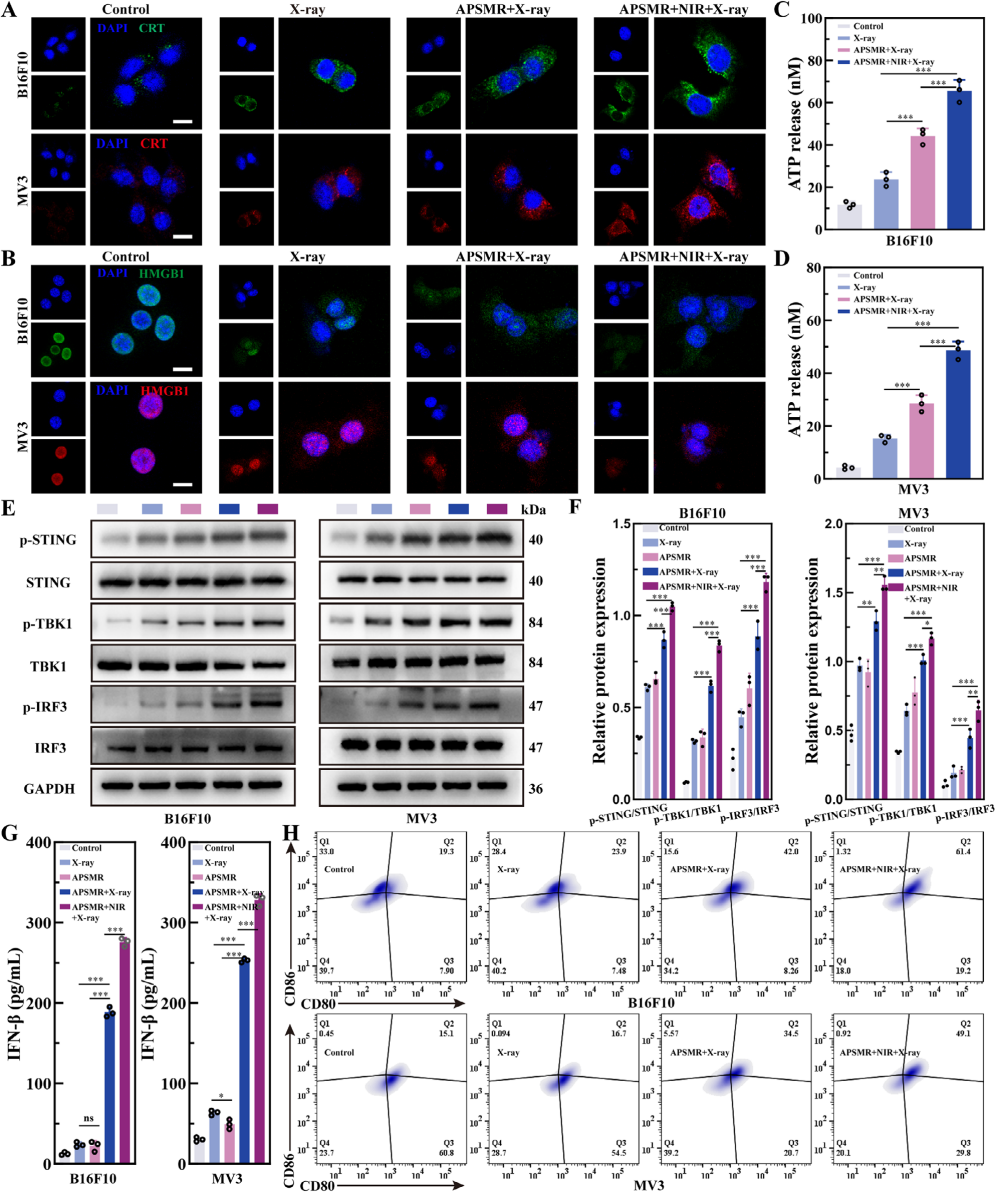
APSMR elicits robust ICD and STING activation. A) Immunofluorescence images of CRT expression in B16F10 and MV3 cells after various treatments (scale bar: 10 µm). B) Immunofluorescence images of HMGB1 expression in B16F10 and MV3 cells after various treatments (scale bar: 10 µm). C,D) ATP release levels in the culture medium of B16F10 and MV3 cells after various treatments. E) The expression levels of cGAS‐STING pathway marker proteins in THP1 cells following co‐incubation with B16F10 and MV3 melanoma cells. F) Statistical analysis of p‐STING, p‐TBK1, p‐IRF3 levels after various treatments. G) ELISA analysis of IFN‐β levels in tumor tissues after different treatments. H) Flow cytometry analysis of CD11c⁺CD80⁺CD86⁺ expression in BMDCs after co‐incubation with B16F10 or MV3 cells. Data are presented as mean values±SD. *p < 0.05, **p < 0.01, ***p < 0.001.

We investigated the immunostimulatory potential of APSMR nanoparticles by examining changes in ICD markers (CRT, HMGB1, and ATP) in B16F10 and MV3 cells. The CRT immunofluorescence assay (**Figure** **6**A) showed that predominantly perinuclear CRT expression. Compared with the RT group, the APSMR+RT group displayed stronger CRT fluorescence on the cell membrane, accompanied by more distinct cell contours. Notably, the APSMR+RT+NIR group exhibited the strongest fluorescence intensity and the most clearly defined cell outlines. Additionally, HMGB1 expression (Figure 6B) was mainly localized in the nucleus of untreated cell; however, following treatment, nuclear fluorescence diminished while weak cytoplasmic fluorescence became apparent. Moreover, as shown in Figure 6C,D, the APSMR+RT+NIR group exhibited the highest extracellular ATP levels in both cell lines, surpassing all other groups. Although ATP release was lower in the APSMR+RT group than in APSMR+RT+NIR, it remained higher than in the RT group. In summary, these results indicate that APSMR nanoparticles elicit a more robust ICD response, evidenced by enhanced CRT membrane expression and increased HMGB1 and ATP release.

We next evaluated whether APSMR nanoparticles enhance cGAS‐STING pathway activation via Western blot analysis. After co‐culturing THP1 cells with B16F10 and MV3 cells, the phosphorylated levels of STING, TBK1, and IRF3 in the APSMR+RT and APSMR+RT+NIR groups were significantly higher than that in the RT group (Figure 6E,F). In addition, IFN‐β concentrations in the co‐culture supernatant were markedly higher in both the APSMR+RT and APSMR+RT+NIR groups than in the RT group, with the combined therapy showing the most pronounced increase (Figure 6G). Finally, flow cytometry analysis (Figure 6H) demonstrated a sequential increase of mature DCs (CD11c^+^CD80^+^CD86^+^) in the RT, APSMR+RT, and APSMR+RT+NIR groups, underscoring the substantial potential of APSMR nanoparticles to synergize radiation and immunotherapy. These findings suggest that APSMR nanoparticles effectively activate the cGAS‐STING pathway and trigger IFN‐β release, thereby amplifying RT's immunostimulatory effects and facilitating antigen cross‐presentation by DCs.

### 2.8 Biotoxicity and Radiosensitization Efficacy Evaluation In Vivo

**Figure 7:**
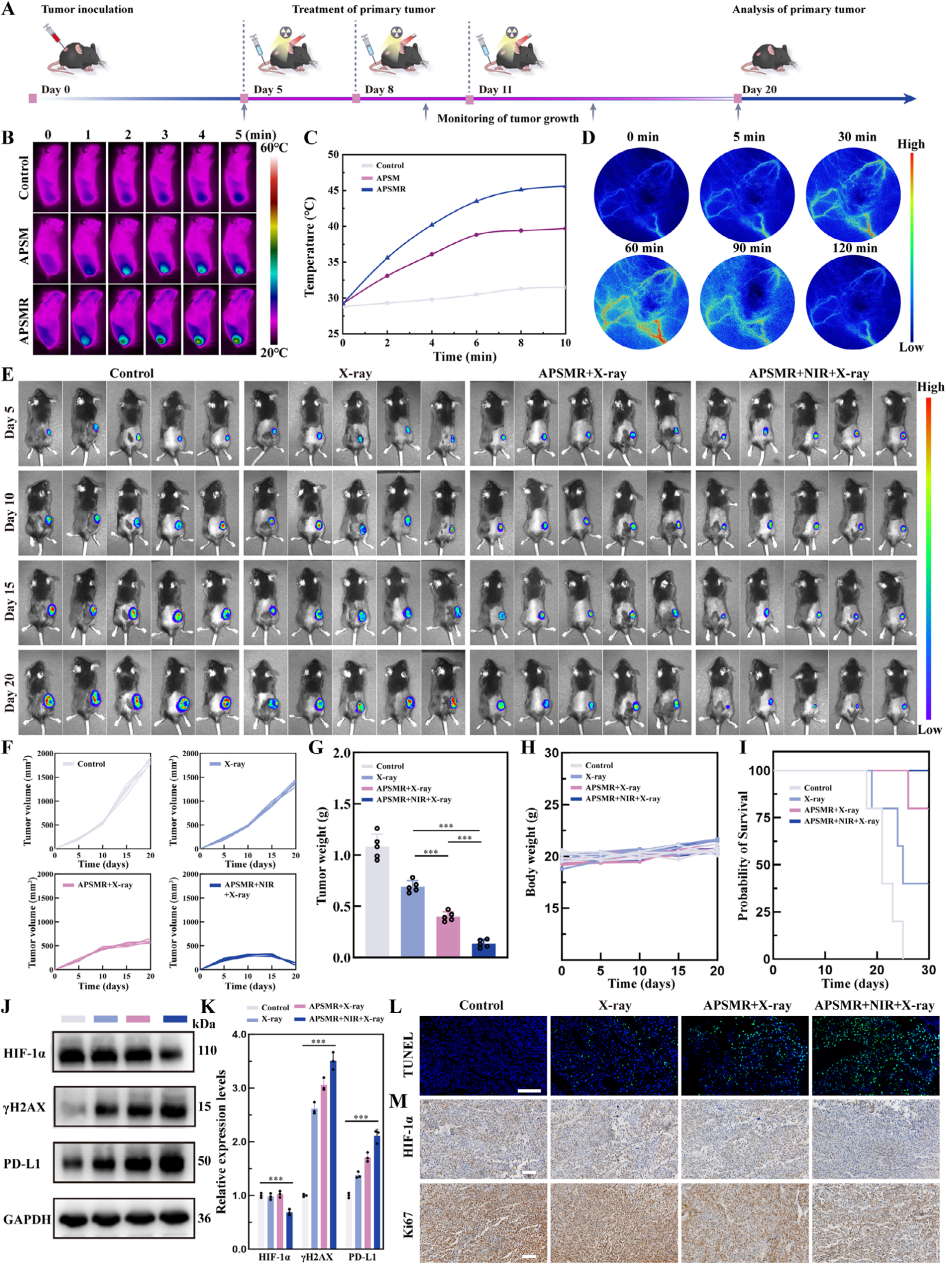
In vivo treatment with APSMR nanoparticles in the mouse tumor model. A) Treatment scheme for APSMR in the mouse tumor model. B) Infrared imaging of B16F10 tumor‐bearing mice post‐1064 nm laser treatment (0.5 W cm^−^^2^, 5 min). C) Photothermal temperature curves at various time points. D) Laser speckle imaging of blood flow signals in tumor tissue at various time points post‐APSMR nanoparticles treatment. E) Fluorescence imaging of B16F10‐Luc tumor‐bearing mice at multiple time points post‐treatment. F) Tumor growth curves across different treatment groups. G) Tumor weight of excised tumors across different treatment groups. H) Body weight curves of tumor‐bearing mice across various treatment groups over time. I) Survival curves of tumor‐bearing mice following various treatments. J) Western blot analysis of HIF‐1α, γH2AX, and PD‐L1 protein levels in tumor samples across different treatment groups. K) Statistical analysis of HIF‐1α, γH2AX, and PD‐L1 levels post‐treatment. L) TUNEL fluorescence staining images of tumor tissues across different treatment groups (scale bar: 100 µm). M) Immunohistochemical staining of HIF‐1α and Ki67 in tumor tissues post‐treatment (scale bar: 100 µm). Data are presented as mean values±SD. *p < 0.05, **p < 0.01, and ***p < 0.001.

Before conducting experiments in tumor‐bearing animal models, we performed hemolysis tests, which demonstrated extremely low hemolysis rates at concentrations up to 400 µg mL^−1^ (Figure S13, Supporting Information), confirming excellent blood compatibility. Furthermore, H&E staining of major organs revealed no appreciable tissue injury or inflammatory infiltration (Figure S14, Supporting Information). We also conducted a panel of hematological and serum biochemistry tests (Figure S15, Supporting Information), including AST, ALT, TBIL, BUN, CREA, WBC, RBC, and PLT. Although some fluctuations were observed, all indices' values remained largely comparable to those of the control group. In conclusion, APSMR nanoparticles exhibit favorable biosafety. **Figure** **7**A presented a schematic overview of the experimental design for the in vivo. To investigate the distribution of nanoparticles within tumor tissues, we used in vivo imaging to assess APSMR accumulation (Figure S16, Supporting Information). The Cy5 fluorescence signal in the tumor region was stronger in the APSMR group than in the APSM group, confirming APSMR's robust tumor‐targeting capabilities. Building on its favorable biosafety and targeting features, we then used an infrared thermal camera to record photothermal images and temperature changes during laser irradiation (Figure 7B,C). While the tumor temperature in the PBS group remained essentially unchanged, the APSM and APSMR groups demonstrated a rapid increase to 39.7 °C and 45.6 °C, respectively. Notably, compared with the APSM, the APSMR group displayed superior photothermal performance, likely due to RGD‐mediated targeting of αvβ3 integrin‐rich tumor cell membranes, thereby facilitating enhanced nanoparticle uptake and internalization. As shown in Figure 7D, laser speckle imaging revealed increased blood flow signals after only 5 min at 43 °C, with a significant improvement by 30 min. This finding suggests that HT effectively alleviates tumor hypoxia, potentially enhancing radiation‐sensitizing effects.

As shown in Figure 7E,F, APSMR nanoparticles displayed a cascading radiation‐sensitizing effect under both X‐ray and 1064 nm laser irradiation, leading to pronounced tumor growth inhibition. On day 10, tumor fluorescence areas did not differ appreciably among the groups. However, on days 15 and 20, in vivo imaging revealed a smaller tumor fluorescence area in the APSMR+RT group relative to controls, and the APSMR+RT+NIR group exhibited the most substantial reduction. These observations suggest that APSMR effectively suppresses melanoma growth under combined X‐ray and 1064 nm laser treatment. Tumor tissues were collected and weighed from euthanized mice (Figure 7G), revealing a progressive decrease in tumor weight across all treatment groups. Additionally, Figure 7H exhibited no significant differences in average body weight over the course of the treatment period, indicating that the tumor model, nanoparticle formulation, and laser irradiation exerted minimal side effects on the animals. The survival curves (Figure 7I) indicated no fatalities in the combined treatment group, which experienced significantly prolonged survival relative to other cohorts. Overall, these initial studies confirm that APSMR nanoparticles possess both radiation‐sensitizing and anti‐melanoma properties. Western blot analysis (Figure 7J,K) further revealed that HIF‐1α expression was markedly reduced in the combined treatment groups compared with the control, indicating that HT alleviated tumor hypoxia and thereby enhancing RT efficacy. Moreover, relative to the control group, PD‐L1 protein expression significantly increased after radiation treatment, with the highest increase observed in the combined RT and HT group. The TUNEL assay (Figure 7L) showed the strongest green fluorescence in the combined treatment group, indicating severe cellular damage. HIF‐1α immunohistochemistry further confirmed that HT alleviated tumor hypoxia (Figure 7M). Finally, the proliferation marker Ki67 was lowest in the combined therapy group (APSMR+RT+NIR), suggesting that this treatment strategy suppresses melanoma cell proliferation, most likely by causing DNA damage.

### 2.9 Systemic Antitumor Immune Response

**Figure 8:**
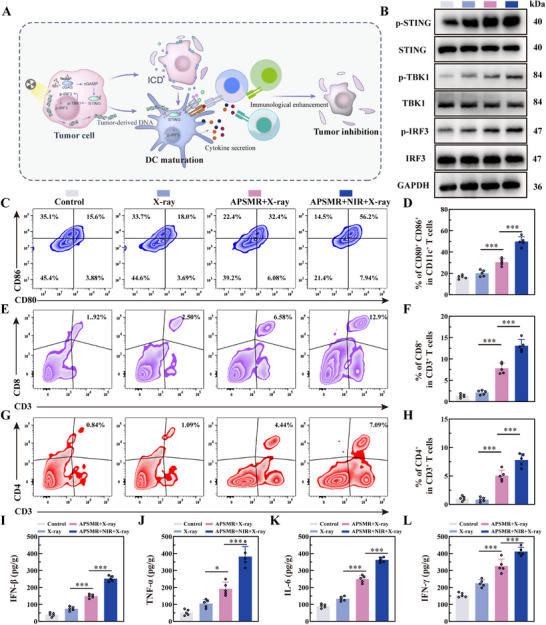
Systemic anti‐tumor immune response. A) Illustration depicting the treatment protocol for B16F10 tumor‐bearing mice. B) Western blot analysis of key proteins in the cGAS‐STING pathway. C) Flow cytometry analysis of mature DC cells (CD11c⁺CD80⁺CD86⁺) percentages in the lymph nodes across various treatment groups. D) Statistical analysis of DC cell proportions in lymph nodes across various treatment groups. E) Flow cytometry analysis of the percentage of CD3^+^ CD8^+^ T cells in the tumors of mice treated with different groups. F) Statistical analysis of the proportion of CD3^+^ CD8^+^ T cells in the tumors of mice treated with different groups. G) Flow cytometry analysis of CD3⁺ CD4⁺ T cell percentages in tumors across various treatment groups. H) Statistical analysis of CD3⁺CD4⁺T cell proportions in tumors across various treatment groups. I–L) ELISA analysis of IFN‐β, IL‐6, TNF‐α, and IFN‐γ levels in tumor tissues across different treatment groups. Data are presented as mean values±SD. *p < 0.05, **p < 0.01, and ***p < 0.001.

To investigate the antitumor vaccine response amplified by the combination of HT and RT, we further examined the immune responses following treatments in each group of mice. **Figure** **8**A presented a schematic illustration of the immunomodulatory mechanism of APSMR nanoparticles. To elucidate this mechanism, we first explored the activation status of the cGAS‐STING signaling pathway. As shown in Figures 8B and S17 (Supporting Information), the key active proteins in this pathway were progressively upregulated, with the differences reaching statistical significance. Next, we examined whether APSMR could promote the maturation of DCs in the inguinal lymph nodes. As presented in Figure 8C, the proportion of mature DCs (CD11c⁺CD80⁺CD86⁺) in the APSMR+RT+NIR group reached ≈56.2%, significantly higher than the 15.6% observed in the control group. The DC maturation rates in the RT and APSMR+RT groups were 18% and 32.4%, respectively, indicating that nanoparticles combined with treatment induced DC maturation. We then assessed the effect of DC maturation on the infiltration of CD8^+^ T cells and CD4^+^ T cells into the primary tumor. Flow cytometry analysis (Figure 8E–H) revealed that the APSMR+RT+NIR treatment drove a pronounced recruitment of T cells into the tumor microenvironment, with CD8⁺ and CD4⁺ populations expanding by ≈12.6‐and 7.6‐fold over controls, respectively. By comparison, APSMR + RT alone induced more moderate, yet still significant, increases of 7.6‐fold for CD8⁺ T cells and 4.8‐fold for CD4⁺ T cells. The moderate infiltration observed in the APSMR+RT group may be attributed to the induction of ICD and Mn^2+^ stimulation of the cGAS‐STING pathway, which triggers an immune‐stimulatory response. To further validate these findings, immunofluorescence analysis of tumor tissues (Figures S18 and S19, Supporting Information) showed that the APSMR+RT+NIR group had the strongest CD8⁺ and CD4⁺ fluorescence signals. Additionally, ELISA was employed to assess the expression of immune‐related cytokines in tumors. As depicted in Figure 8I–L, relative to the control group, cytokine levels increased in the APSMR+RT group, while the APSMR+RT+NIR group displayed the most pronounced elevation in IFN‐β, IL‐6, TNF‐α, and IFN‐γ. Overall, these results confirmed that APSMR nanoparticles triggered cascading immunostimulatory effects, significantly augmenting the RT‐mediated vaccine response.

### 2.10 Inhibition of Metastasis and Long‐Term Immune Memory

**Figure 9:**
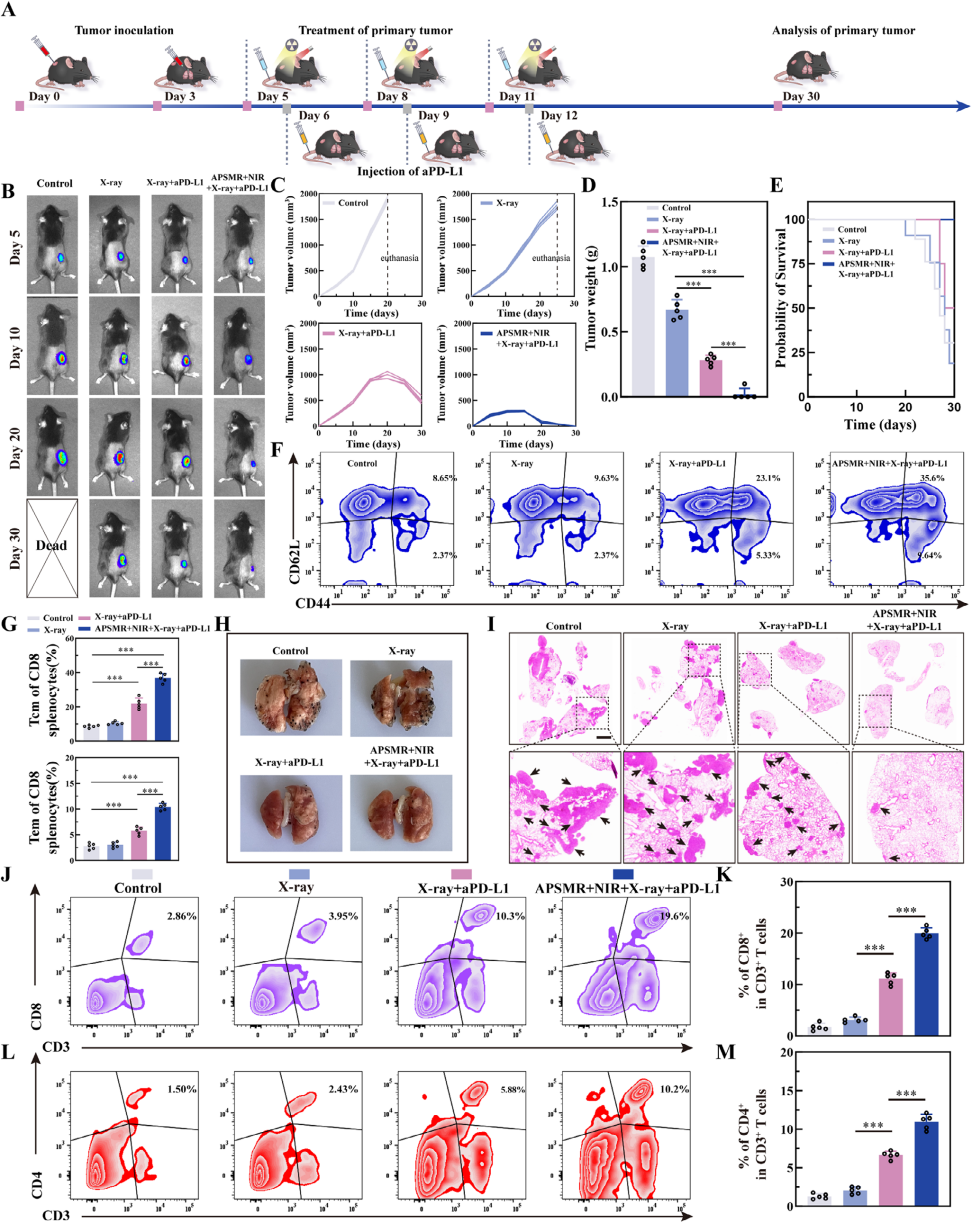
Inhibition of metastasis and long‐term immune memory. A) Experimental design diagram for combining APSMR with anti‐PD‐L1 therapy in tumor‐bearing mice. B) Fluorescence images of B16F10‐Luc tumor‐bearing mice after different treatments. C) Tumor growth curves with time under various treatments. D) Tumor weight after different treatment. E) Survival curves of mice after different treatment groups. F,G) Proportions of Tcm and Tem cells in the spleens across various treatment groups, along with statistical analysis. H) Representative digital images of lung tissues in different groups. I) H&E staining images of lung tissues (black arrows: tumor cells, scale bar: 1 mm). J) Flow cytometry analysis of CD3⁺ CD8⁺ T cell percentages in tumors from various treatment groups. K) Statistical analysis of CD3⁺ CD8⁺ T cell proportions in tumors from various treatment groups. L) Flow cytometry analysis of CD3⁺ CD4⁺ T cell percentages in tumors from various treatment groups. M) Statistical analysis of CD3⁺ CD4⁺ T cell proportions in tumors from various treatment groups. Data are presented as mean values±SD. *p < 0.05, **p < 0.01, and ***p < 0.001.

Building upon the above findings, we explored the therapeutic efficacy of APSMR nanoparticles combined with anti‐PD‐L1 I both primary and metastatic tumors. The experimental design was depicted in **Figure** **9**A. As shown in Figure 9B, by day 30, the RT group exhibited limited inhibition of tumor growth compared to the control group. In contrast, the RT+aPD‐L1 group demonstrated significant tumor suppression, although residual tumor was still observed. Notably, the APSMR+RT+NIR+aPD‐L1 group showed almost no visible tumor tissue. Tumor volume (Figure 9C) monitoring revealed a marked reduction beginning on day 20 in both the RT+aPD‐L1 and APSMR+RT+NIR+aPD‐L1 groups, with the latter showing the greatest tumor inhibition. As illustrated in Figure 9D, the average tumor weight progressively decreased across the treated groups. Survival analysis (Figure 9E) additionally showed no fatalities in the combination therapy group, whereas varying levels of mortality were noted in the other three groups. Figure 9J,L depicted CD8⁺ and CD4⁺ T cell infiltration in tumor tissues. Flow cytometry analysis revealed that the APSMR+RT+NIR+aPD‐L1 group exhibited the highest proportion of T cell infiltration compared with the other three groups, with statistically significant differences (Figure 9K,M). Consistent with these findings, key immune cytokines (Figure S20, Supporting Information) were expressed at their highest levels in the combination therapy group, further substantiating a robust immune response. To assess the anti‐metastatic potential of each treatment strategy, we evaluated lung tumor metastases using a pulmonary metastasis model. Lung images (Figure 9H) revealed numerous black metastatic nodules in the control and RT groups. While the RT+aPD‐L1 group showed a reduction in the number of metastases, scattered black foci were still evident. In contrast, no visible metastatic lesions were detected in the APSMR+RT+NIR+aPD‐L1 group. To further confirm these findings, H&E staining of lung tissues was performed (Figure 9I). The results showed extensive tumor cell infiltration in the control and RT groups, whereas fewer tumor cells were observed in the RT+aPD‐L1 group. In the APSMR+aPD‐L1 combination group, only sparse, isolated tumor cells were detected in the lung tissue. Given that APSMR nanoparticles induced a strong immune response, we further assessed the activation of immune memory by analyzing the proportion of effector memory T cells (Tem, CD62L^−^CD44^+^) and central memory T cells (Tcm, CD62L^+^ CD44^+^) in the spleen (Figure 9F). The proportion of Tem and Tcm cells in the APSMR+RT+NIR+aPD‐L1 group exceeded that in controls, indicating the establishment of robust immune memory (Figure 9G). In conclusion, these results suggest that APSMR nanoparticles effectively remodel the immunosuppressive tumor microenvironment, trigger strong immune responses, induce long‐term immune memory, and synergize with immune checkpoint blockade to potentially eradicate residual tumors.

## 3 Conclusion

In summary, this study developed an anisotropic Au‐Pd heterostructure nanosystem (APSMR) designed to enhance radioimmunotherapy in melanoma treatment. The results demonstrated that APSMR effectively overcame the inherent radioresistance of melanoma and significantly amplified the abscopal effect, which is critical for the complete eradication of systemic tumors. Specifically, the Au component, enhanced local radiation energy deposition, thereby effectively mitigating the radiation resistance induced by melanin accumulation. The Pd nanoparticles with antenna‐like heterostructures exhibited exceptional photocatalytic and photothermal conversion efficiencies under 1064 nm laser irradiation. Moreover, HT of tumor tissues to 43 °C was identified as optimal for radiosensitization, as this temperature alleviated hypoxia, inhibited DNA damage repair mechanisms, and arrested the cell cycle at the radiosensitive G2 phase, collectively improving the therapeutic efficacy of radiation. Regarding immune modulation, APSMR induced pronounced ICD, characterized by the externalization of CRT on tumor cell surfaces and the release of HMGB1 and ATP. Additionally, Mn^2^⁺ ions released from the APSMR nanoparticles activated the cGAS‐STING signaling pathway and stimulating type I interferon production. These events synergized with ICD to elevate the overall radioimmunotherapeutic efficacy, substantially promoting DC maturation and enhancing the infiltration of CD8⁺ and CD4⁺ T cells into tumor tissues. Furthermore, it was observed that APSMR‐mediated RT and HT upregulated PD‐L1 expression in melanoma. Consequently, combination therapy with anti‐PD‐L1 antibodies demonstrated notable synergistic effects, effectively inhibiting the growth of primary and metastatic tumors and inducing long‐term immune memory. Overall, the APSMR nanosystem presents a novel multifunctional and synergistic therapeutic strategy, providing significant innovative perspectives for radioimmunotherapy and offering pivotal insights for the future development of adjuvant‐free vaccine carriers.

## 4 Experimental Section

### Materials

All chemicals utilized in the experiments were of analytical grade and required no additional purification. AA, NaOL, HAuCl_4_·3H_2_O, NaBH_4_, CTAB, CPC, Na_2_PdCl_4_, TEOS, NHS, EDS, MB, DMPO, TEMP, AgNO_3_, MnCl_2_ **·**4H_2_O, NH4Cl, NaOH, Ethanol, Ammonia solution, were purchased from Aladdin (Shanghai, China). Calcein AM/PI Cell Viability Assay Kit, Annexin V‐FITC/PI Detection Kit, JC‐1 Apoptosis Detection Kit, Cell damage agent test kit by comet electrophoresis were purchased from Nanjing KeyGen Biotech Inc. DCFH‐DA were obtained from Sigma‐Aldrich (Shanghai, China). ATP assay kits were purchased from Beyotime Institute of Biotechnology. Anti‐PD‐L1 antibody, Anti‐HMGB1 antibody, Anti‐Calreticulin antibody were purchased from Abcam. Anti‐STING antibody, Anti‐Phospho‐STING antibody, Anti‐TBK1/NAK antibody, Anti‐Phospho‐TBK1/NAK antibody, Anti‐IRF‐3 antibody, Anti‐Phospho‐IRF‐3 antibody, Anti‐Phospho‐Histone H2AX antibody were purchased from Cell Signaling Technology Inc. CD45 Monoclonal Antibody, CD3 Monoclonal Antibody, CD8 Monoclonal Antibody, CD4 Monoclonal Antibody, CD11c Monoclonal Antibody, CD80 Monoclonal Antibody, CD86 Monoclonal Antibody, CD62L Monoclonal Antibody, CD44 Monoclonal Antibody were purchased from Thermo Fisher Scientific Inc. ELISA kits for IFN‐β, TNF‐α, IFN‐γ, and IL‐6 analysis were purchased from Proteintech Group and Thermo Fisher Scientific Inc.

### Instrumentation

Transmission electron microscopy (TEM, FEI Tecnai T20), scanning transmission electron microscopy (STEM, FEI TF20) were used to observe the surface morphology and internal structure of the nanoparticles. X‑ray photoelectron spectroscopy (XPS, ESCALAB 250Xi) was performed to characterize the elements present and their oxidation states. UV–vis spectroscopy (Cary 60) was employed to measure the absorption spectra of the nanoparticles. Dynamic light scattering (DLS, DynaPro NanoStar) was used to analyze and record the hydrodynamic diameter of the nanoparticles. Electron spin resonance spectroscopy (ESR, Bruker A300) was employed to identify the ROS generated. Zeta potential analysis (NanoPlus) was performed to measure the zeta potential. The mice were subjected to treatment using a small animal X‐ray irradiator (X‐RAD225, Precision X‐Ray). Flow analysis was performed by flow cytometry (FACSCanto II, BD). The fluorescent images were captured by confocal laser scanning microscopy (CLSM, STELLARIS 5, Leica). Blood flow pictures were captured by laser speckle imaging system (FLSI III, RWD).

### Cell Culture

HaCaT and MV3 cells were obtained from the Shanghai Cell Bank (Chinese Academy of Sciences) and maintained in DMEM containing 10% fetal bovine serum (FBS). B16F10 and B16F10‑Luc murine melanoma lines and THP‑1 cells (Shanghai Fuheng Biotechnology Co., Ltd.) were cultured in RPMI‑1640 with 10% FBS; THP‑1 medium was further supplemented with 0.05 mm β‑mercaptoethanol.

### Animals

For animal model establishment, female C57BL/6J mice, aged six weeks, were procured from GemPharmatech Co., Ltd. The mice were housed under SPF conditions at Xuzhou Medical University with sterilized food and water provided ad libitum. A subcutaneous tumor model was established by injecting 5 × 10⁶ B16F10 cells into the right posterior flank of the mice. Additionally, a lung metastasis model was initiated via the intravenous administration of 2 × 10^6^ B16F10 cells into the mice. The Ethics Committee of Xuzhou Medical University reviewed and approved all animal experiments (approval No. 202209S085).

### Preparation of Au NRs

A seed solution was first generated by combining 5 mL of 0.5 mm HAuCl_4_ with an equal volume of 0.2 M CTAB. 0.6 mL of freshly prepared 0.01 M NaBH_4_ was swiftly added under vigorous stirring at 1200 rpm, inducing a color change to brownish‐yellow. The solution was then incubated at 30 °C for 2 h before being stored. In the next step, a 250 mL flask was used to dissolve 3.6 g of CTAB and 0.494 g of NaOL, followed by the addition of 9.6 mL of 4 mm AgNO_3_ and 100 mL of 1 mM HAuCl_4_. This mixture was maintained at 30 °C for 15 min and stirred at 600 rpm for 90 min. Following this, 0.32 mL of the pre‐aged seed solution was injected, and the mixture was maintained for 12 h to facilitate nanorod growth. Collect the resulting Au nanorods by centrifugation at 8000 rpm, discard the supernatant, redispersed the pellet in 20 mL ultrapure water via sonication, and store at 4 °C until further use.

### Preparation of AP Heterostructures

A 20 mL CPC solution (10 mm) was brought to 65 °C, then 200 µL of 10 mm Na_2_PdCl_4_ and 2 mL of Au NRs were introduced. After rapid injection of 400 µL freshly prepared 100 mm AA, the mixture was agitated vigorously for 2 min and allowed to react for 30 min. The precipitated product was collected by centrifugation at 8000 rpm for 15 min for further use.

### Preparation of APSMR Nanoparticles

AP heterostructures (1 mg) were dispersed in a mixture of 1 mL 5 mm CTAB, 7 mL ultrapure water, and 100 µL 0.1 m NaOH, then gently stirred at 30 °C. Over 48 h, 30 µL portions of 0.9 M TEOS were added every 45 min (three additions total). The suspension was centrifuged at 8000 rpm to harvest the precipitate, which was resuspended with 10 mg MnCl_2_·4H_2_O, 40 mg NH_4_Cl, and 2 mL water plus 20 µL NH_3_·H_2_O. After stirring for 3 min, the mixture was sealed in a 5 mL autoclave and reacted at 160 °C for 4 h. The product was washed, centrifuged, and redispersed in 2 mL water. For surface functionalization, 2 mg EDC was added and stirred 15 min, then 2 mg NHS was introduced and stirred at room temperature for 4 h. Next, 1 mg RGD peptide was added, stirred 2 h, and the reaction allowed to proceed overnight. Finally, APSMR nanoparticles were purified, redispersed in ultrapure water, and stored at 4 °C.

### In Vitro Degradation of APSMR

100 µg of APSMR nanoparticles were dissolved in 1 mL of ultrapure water at pH 7.4 or in 1 mL of ultrapure water containing 5 mm GSH at pH 6.5, and incubated overnight. On the following day, 1 mL of the APSMR solution was transferred to a quartz cuvette for DLS analysis to measure the hydrodynamic diameter of the nanoparticles.

### Detection of Reactive ROS

In accordance with the aforementioned degradation reaction system, the APSMR aqueous solution, after degradation, was supplemented with H_2_O_2_ to a final concentration of 1 mm, MB at 10 µg mL^−1^, and NaHCO_3_ at 25 mm. 100 mm DMPO was used as a trapping agent to •OH. For the detection of ¹O_2_, three groups were prepared: PBS, APSMR, and APSMR+NIR. The laser group solution was irradiated with a 1064 nm laser, followed by detection using 10 mm TEMP as a trapping agent for ¹O_2_.

### Photothermal Properties of APSMR

APSMR suspensions were prepared at 0, 25, 50, and 100 µg mL^−1^ and irradiated with a 1064 nm laser for 10 min, with temperature changes recorded by infrared thermography. The photothermal stability of the 100 µg mL^−1^ APSMR solution was tested through 6 cycles, each involving heating and natural cooling periods, and a photothermal stability curve was plotted.

### Cytotoxicity Assay

HaCaT, B16F10, and MV3 cells were treated with culture medium supplemented with APSMR nanoparticles at concentrations ranging from 0 to 200 µg mL^−1^ and incubated for 24 h. Cell viability was evaluated using the CCK‐8 assay. Additionally, B16F10 and MV3 cells were exposed to different concentrations of the nanoparticles for 12 h, followed by 1 h irradiation with a 1064 nm laser at 43 °C. Immediately thereafter, cells received a radiation dose of 4 Gy and were further cultured for 48 h before cell viability was reassessed using the CCK‐8 assay.

### Cellular Uptake Experiment

To prepare FITC‐labeled APSM and APSMR nanoparticles, 0.5 mg of FITC was added to 1 mg mL^−1^ aqueous solutions of APSM or APSMR nanoparticles. The mixtures were subjected to ultrasonic dispersion followed by continuous stirring for 24 h. The resulting FITC‐labeled nanoparticles (APSMR‐FITC) were collected via centrifugation and resuspended in culture medium to a final concentration of 100 µg mL^−1^. B16F10 and MV3 cells were then incubated with 1 mL of this nanoparticle suspension for 5 min, 1 h, 3 h, or 6 h. After incubation, nuclei were counterstained with DAPI for 2 min, unbound particles were removed via PBS washes, and cellular internalization was assessed by CLSM.

### Multicellular Spheroid Model Assay

B16F10 and MV3 cells (1 × 10^4^) were seeded into low‐attachment 24‐well plates and cultured for 10 days. Nanoparticles APSM‐FITC and APSMR‐FITC nanoparticles were then added at 100 µg mL^−1^ and incubated for 6 h. The spheroids were transferred to confocal dishs and the Z‐axis was adjusted on the confocal microscope to observe the fluorescence changes within the cells, assessing the uptake of the nanoparticles.

### Detection of Intracellular ROS Generation

B16F10 and MV3 cell lines were categorized into four experimental cohorts: PBS‐only, RT‐alone, APSMR+RT, and APSMR+NIR+RT. Each group had cells treated with a 1 mL dose of 100 µg mL^−1^ nanoparticle formulation for 12 h, followed by designated treatments. In the NIR laser treatment cohort, the cells underwent 60 min of irradiation with a 1064 nm laser (0.5 W cm^−2^), maintaining a constant temperature of 43 °C as confirmed via infrared thermal imaging. The RT group received a 4 Gy exposure. Post‐treatment, all groups were subjected to a 60‐min incubation with 5 µm DCFH‐DA, followed by multiple PBS washes. Fluorescence microscopy facilitated the visualization and documentation of cellular responses. For flow cytometric analysis, the procedure mirrored the aforementioned steps until post‐DCFH‐DA incubation, where cells were then detached using trypsin, harvested, and subjected to flow cytometry to assess ROS levels.

### In Vitro Evaluation

B16F10 and MV3 cells were seeded into the appropriate culture plates and divided into four groups: PBS, RT, APSMR+RT, and APSMR+NIR+RT. Nanoparticles were applied at 100 µg mL^−1^, and the cells were irradiated with a 1064 nm laser (0.5 W cm^−2^) for 60 min, with the RT group receiving a dose of 4 Gy. Following 48 h of culture, cell damage was quantified by measuring mitochondrial membrane potential, performing live/dead staining, and conducting Annexin V‐FITC/PI assays.

### Colony Formation Assay

B16F10 and MV3 cells were seeded into 6‐well plates and allowed to adhere for 24 h before receiving treatments according to the designated experimental groups. After a 10‐day incubation, colonies were fixed and stained using crystal violet. The colonies were then counted, and colony formation rates were determined by comparing with control groups. Radiation Sensitization Rate = (Experimental Group Colonies/Control Group Colonies) × 100%.

### Comet Assay

B16F10 and MV3 cells were divided into four groups: PBS, RT, APSMR+RT, and APSMR+NIR+RT, with each group receiving 100 µg mL^−1^ of APSMR nanoparticles. After 24 h incubation, cells were collected, and adjusted to a final concentration of 1 × 10^6^ cells mL^−1^. Subsequently, cells were subjected to agarose gel coating, lysis for 2 h, DNA alkaline unwinding for 30 min, electrophoresis for 20 min, and PI staining for 10 min. DNA damage was evaluated using fluorescence microscopy and CASP software.

### γH2AX Analysis

B16F10 and MV3 cell lines were treated following standardized protocols. Post‐treatment, the cells underwent fixation and subsequent permeabilization for a duration of 1 h. An overnight incubation at 4 °C was then performed using a γH2AX monoclonal antibody. Following this, cells were then incubated for 1 h with secondary antibodies tagged with Alexa Fluor 488 and Alexa Fluor 594, and nuclei were subsequently counterstained with DAPI for visualization. The resultant fluorescence was captured and documented using fluorescence microscopy. For the analysis of protein expression, a comprehensive procedure was executed, encompassing cell lysis, extraction of proteins, determination of protein concentrations, electrophoretic separation, membrane transfer, antibody incubation, and imaging. The data obtained from Western blot analyses were subsequently evaluated using ImageJ software.

### DNA Damage Repair Analysis

After a 24 h treatment as described earlier, B16F10 and MV3 cells were collected and subjected to Western blotting to evaluate the levels of key double‐strand break repair proteins (BRCA1, BRCA2, Ku70, Ku80, and Rad51).

### ICD Biomarker Detection

Cells were assigned into four groups: PBS, RT, APSMR+RT, and APSMR+NIR+RT, and treated accordingly. Post‐treatment, primary antibodies against CRT and HMGB1 were applied for 12 h, followed by incubation with Alexa Fluor‐488 and Alexa Fluor‐594 secondary antibodies for 1 h. Cells were then stained with DAPI for 5 min and visualized by CLSM to assess CRT and HMGB1 release. ATP levels were measured by collecting cell supernatant, determining ATP concentration via a standard curve, adding the ATP working solution to the reaction system, and quantifying ATP content using a chemiluminescence analyzer.

### STING Signaling Pathway

Following the initial treatments, B16F10 and MV3 cells were co‐cultured with THP1 cell. THP‑1 cells were subsequently collected for Western blot analysis to assess both total and phosphorylated STING, TBK1, and IRF3 levels. In parallel, the culture supernatants were collected to quantify IFN‐β secretion using an ELISA kit.

### Flow Analysis of BMDCs

The extraction and culture of bone marrow‐derived dendritic cells (BMDCs) were conducted according to protocols described in the literature.^[^ ^48^ ^]^ Cultured BMDCs were co‐cultured with pre‐treated B16F10 and MV3 cells. Afterward, cells were stained on ice for 30 min with anti‐ CD11c, CD80, and CD86 antibodies, analyzed by flow cytometry, and data processed using FlowJo software.

### Hemolysis Test

Red blood cells (RBCs) were separated from whole blood collected from healthy C57BL/6J mice by centrifugation, removing the serum and resuspending the cells in PBS. These RBCs were then incubated with APSMR at 0, 50, 100, 200, or 400 µg mL^−1^ for 2 h at 37 °C. Hemolysis was determined by measuring hemoglobin release at 540 nm on a microplate reader, with PBS and water serving as the negative and positive controls, respectively.

### Comprehensive In Vivo Safety Evaluation

For further safety assessments, four groups of mice (n = 5 per group) were established, receiving intravenous injection of APSMR nanoparticles at 0, 10, 20, and 40 mg kg^−1^. Over a span of six days, three injections were administered bi‐daily. Following 48 h post‐final injection, orbital sinus blood extraction was performed, and the mice were euthanized to collect major organs for subsequent blood tests, biochemical analysis, and histopathological examination.

### Targeting Efficacy in Animal Models

Cy5‐tagged APSM and APSMR nanoparticles (10 mg kg^−1^) were delivered via intravenous injection, and tumor fluorescence was captured at 3, 6, and 12 h post‐injection using an in vivo imaging system to evaluate nanoparticle accumulation and targeting efficiency.

### In Vivo Photothermal Effectiveness Testing

Mice with established subcutaneous tumors were divided into three groups: control (PBS), APSM, and APSMR, and received intravenous injections of 100 µL of either APSM or APSMR nanoparticles (10 mg mL^−1^), with PBS serving as the control. Tumor sites were then irradiated with a 1064 nm laser, and surface temperatures were recorded via infrared thermal imaging to evaluate the nanoparticles' photothermal effect.

### In Vivo Blood Flow Assessment

Tumor‐bearing mice were randomly assigned to three groups: PBS, APSM, and APSMR. The treatment regimen was the same as described above. Laser speckle flow imaging was used to capture and analyze blood flow velocity changes over time (0, 5, 30, 90, 120 min).

### In Vivo Antitumor Effect of APSMR

Tumor bearing C57BL/6J mice were stratified into four groups: control, RT, APSMR+RT, and APSMR+RT+NIR. APSMR nanoparticles were administered intravenously at 10 mg kg^−1^. A radiation dose of 4 Gy was delivered, and tumor temperature was maintained at 43 °C for 1 h using infrared thermal imaging. This treatment regimen was repeated every three days, totaling three sessions. Tumor progression was monitored in real‐time via in vivo fluorescence imaging conducted every 5 days, and both body weight and tumor volume (calculated as length × width^2^/2) were regularly measured. Survival rates were recorded daily. After 20 days, the mice were sacrificed, and tumors were excised, weighed, and analyzed to assess treatment efficacy. Western blot analysis was performed to detect γH2AX, HIF‐1α, PD‐L1, and proteins involved in the cGAS‐STING pathway.

### TUNEL Staining

Tumor sections from each treatment group were first deparaffinized and rehydrated, then rinsed in PBS. Slides were then treated with proteinase K (20 µg mL^−1^) for 20 min, followed by another PBS wash. Next, sections were incubated in equilibration buffer for 20 min. TUNEL labeling was carried out by applying TdT reaction mix and incubating in the dark at 37 °C for 1 h. After two PBS rinses, nuclei were counterstained with DAPI for 5 min, washed once more, and immediately examined by fluorescence microscopy to assess DNA fragmentation.

### In Vivo Immunoenhancement Effect of APSMR

Tumor tissues and lymph nodes from each experimental group were processed by transferring them onto a 300‐mesh cell strainer, followed by gentle homogenization and enzymatic digestion. The cell suspensions were then washed twice with ice‐cold PBS, pelleted by centrifugation (300 g, 5 min), and resuspended at 1 × 10^7^ cells mL^−1^. Subsequently, flow cytometry antibodies targeting CD11c, CD80, CD86, CD3, CD4, and CD8 were added, and the cells incubated in darkness for 30 min. Samples were analyzed by flow cytometry, and data processing was performed using FlowJo software. Concurrently, tumor tissues were collected, serum isolated, and cytokine levels of IFN‐β, IL‐6, TNF‐α, and IFN‐γ were measured using ELISA.

### APSMR Combined with Anti‐PD‐L1 Treatment Study

Three days post the subcutaneous injection of 5 × 10^6^ B16F10‐Luc cells, an additional 2 × 10^6^ cells were administered intravenously to establish a model for assessing systemic therapeutic effects. On day 5, mice were assorted into four groups (n = 5): control, RT, aPD‐L1+RT, and APSMR+RT+NIR+aPD‐L1. Each group received designated treatments; APSMR was administered intravenously at 10 mg kg^−1^, and aPD‐L1 at 5 mg kg^−1^. The tumors were exposed to a 1064 nm laser for 1 h, maintaining the temperature at 43 °C, with the protocol repeating every three days for a total of three sessions. Tumor progression and survival rates were continuously monitored. By day 30, mice were sacrificed, and tumors along with major immune organs were harvested for immune cell profiling, specifically analyzing CD3, CD4, CD8, CD62L, and CD44 expressions. Additionally, lung tissues were excised, imaged, and histologically examined for metastatic lesions via H&E staining.

### Statistical Analysis

The data collected were presented as mean ± standard deviation (SD). Statistical significance was assessed using a one‐way ANOVA, with significance thresholds set at *p < 0.05, **p < 0.01, and ***p < 0.001. GraphPad Prism software version 9.5.0 was employed for all statistical computations. Flow cytometry data were processed with FlowJo version 10.8.1, and ImageJ version 1.4.3.67 was used for the analysis of fluorescent grayscale images.

## Conflict of Interest

The authors declare no conflict of interest.

## Supporting information



Supporting Information

## Data Availability

The data that support the findings of this study are available from the corresponding author upon reasonable request.
