# Supplementary material for: An Anisotropic Gold‐Palladium Heterostructured Nanosystem for Synergistically Overcoming Radioresistance and Enhancing Melanoma Radioimmunotherapy
Source: Adv Sci (Weinh). 2025 Jun 17;12(31):e00492. doi: 10.1002/advs.202500492 (PMC12376628; doi:10.1002/advs.202500492)
Supplement: Supplementary file 1 — Supporting Information [file ADVS-12-e00492-s001.docx]

Supporting Information

**An Anisotropic Gold-Palladium Heterostructured Nanosystem for Synergistically Overcoming Radioresistance and Enhancing Melanoma Radioimmunotherapy**

Cheng Chen, Yuqi Huang, Wandong Wang, Minghao Chao, Weiguo Sun, Yinghui Kong, Guan Jiang^*^, Yong Gao^*^ and Fenglei Gao^*^

C. Chen, W. Sun, Y. Kong

Department of Dermatology

The Affiliated Huaian No. 1 People's Hospital of Nanjing Medical University

Jiangsu 223300, P. R. China

Y. Gao

Department of Oncology

The Affiliated Huaian No. 1 People's Hospital of Nanjing Medical University

Jiangsu 223300, P. R. China

E-mail: hayygaoy@njmu.edu.cn (Y. Gao).

W. Wang, M. Chao, F. Gao

Jiangsu Key Laboratory of New Drug Research and Clinical Pharmacy

Xuzhou Medical University

Jiangsu 221004, P. R. China

E-mail: flgao@xzhmu.edu.cn (F. Gao).

G. Jiang

Department of Dermatology,

Affiliated Hospital of Xuzhou Medical University,

Xuzhou, Jiangsu 221002, People’s Republic of China.

E-mail: dr.guanjiang@gmail.com (G. Jiang).

Y. Huang

Department of Dermatology

The Affiliated Suzhou Hospital of Nanjing Medical University

Jiangsu 215000, P. R. China

W. Wang

Department of Dermatology

University Medical Center Groningen, University of Groningen

Groningen 9713 GZ, the Netherlands


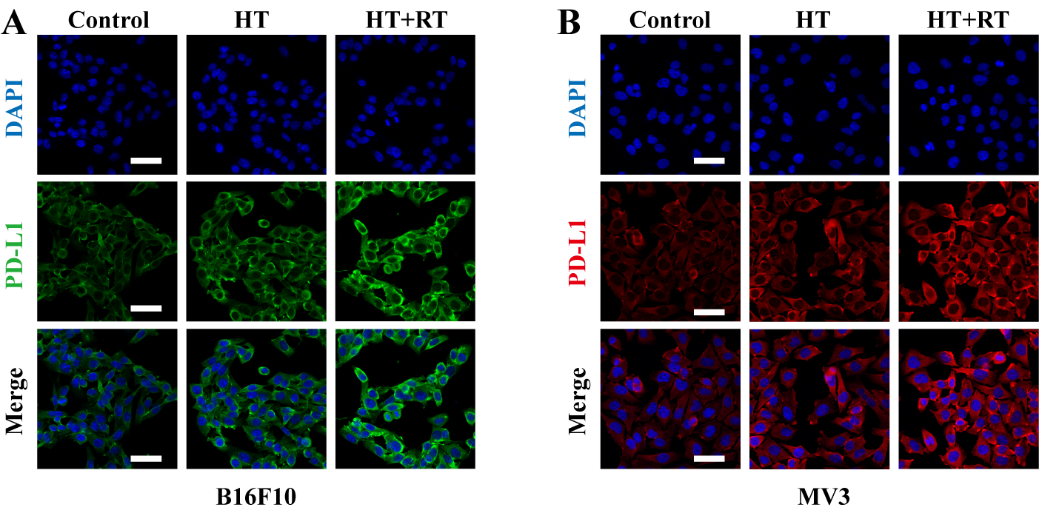


**Figure S1.** (A-B) Immunofluorescence images displaying PD-L1 expression in B16F10 and MV3 cells after HT and RT treatment (HT: 43℃, RT: 4Gy, scale bar: 50 µm).


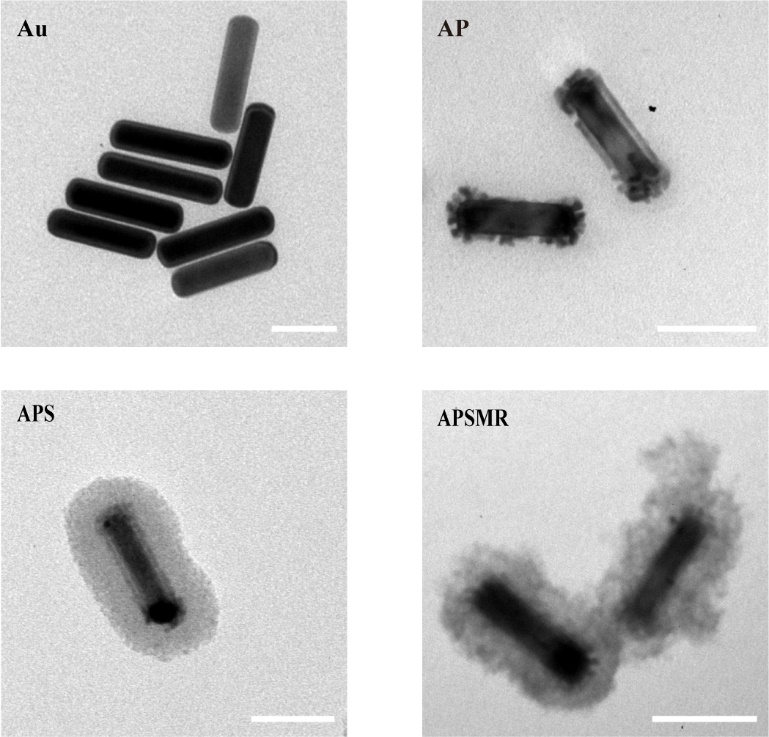


**Figure S2.** TEM images of nanoparticles at each step (scale bar: 50 nm).


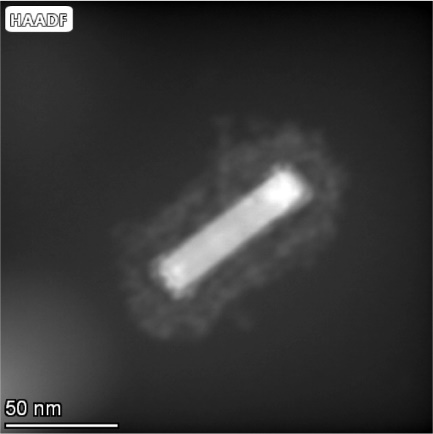


**Figure S3**. STEM images of APSMR nanoparticles.

| **Element** | **Family** | **Atomic Fraction (%)** | **Mass Fraction (%)** |
| --- | --- | --- | --- |
| Au | L | 11.79 | 36.62 |
| Pd | L | 8.62 | 14.47 |
| Mn | K | 32.29 | 27.97 |
| Si | K | 47.29 | 20.94 |

**Figure S4**. EDS analysis of the atomic Fraction and mass fraction of Au, Pd, Mn and Si elements in APSMR.


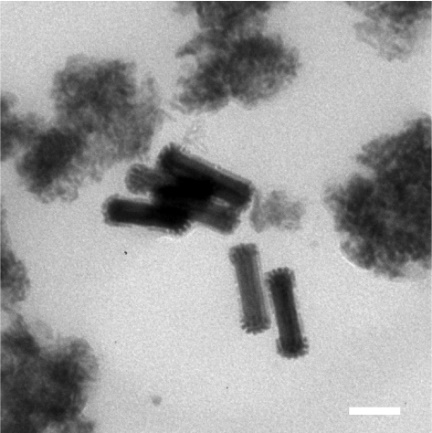


**Figure S5**. A TEM image of APSMR nanoparticles biodegradation after 24 h in a solution mimicking the tumor microenvironment (scale bar: 50 nm).


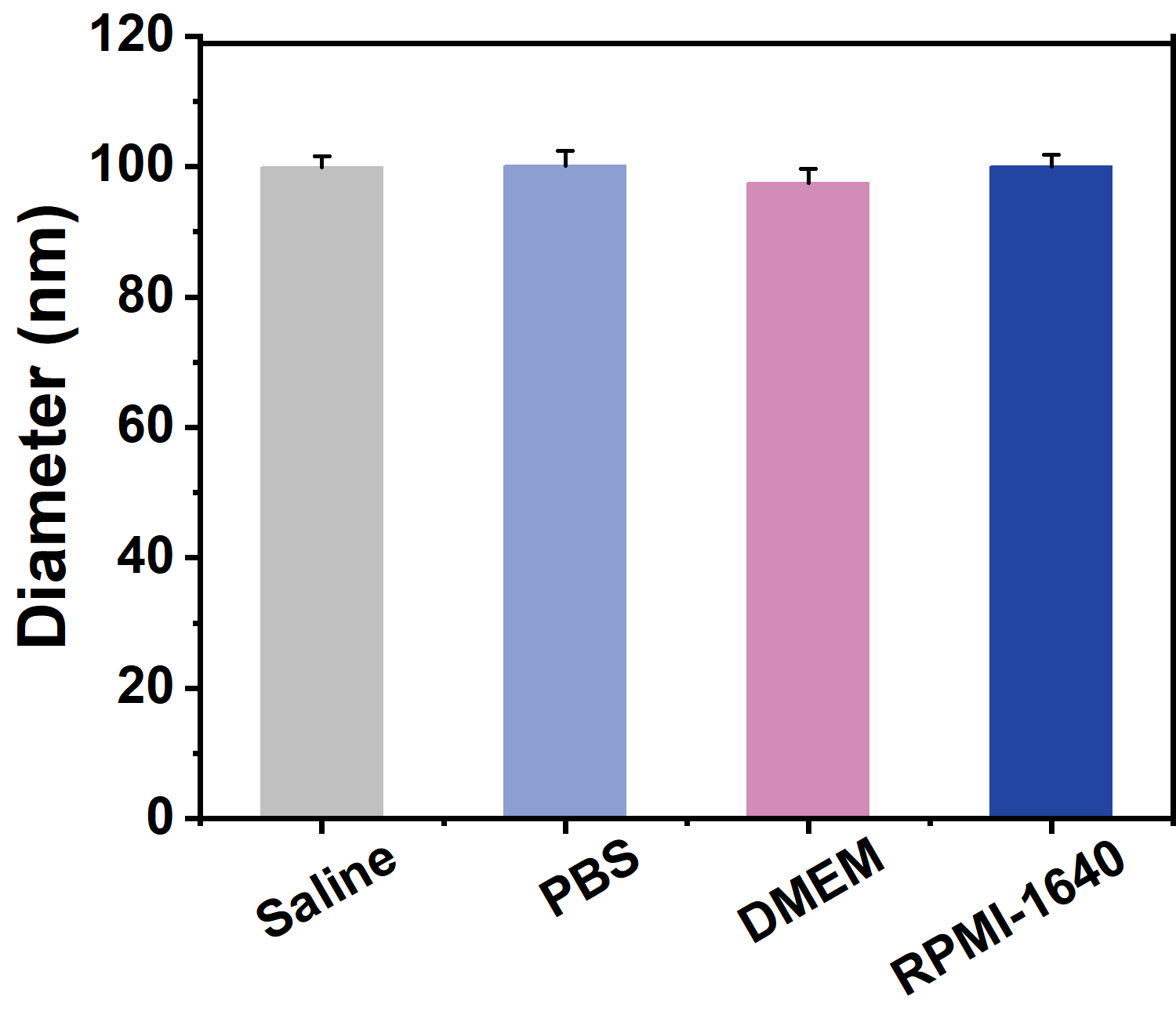


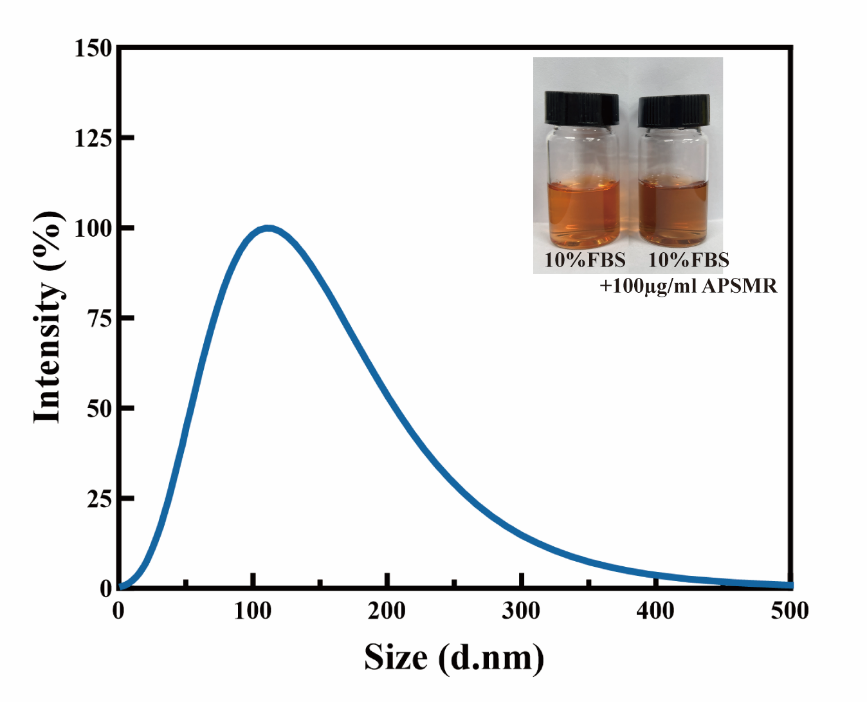
**Figure S6.** The particle size of APSMR after 24 h in saline, PBS, DMEM and RPMI-1640 medium.

**Figure S7.** The particle size of APSMR in 10% fetal bovine serum solution at 37 ℃ for up to 48 h.


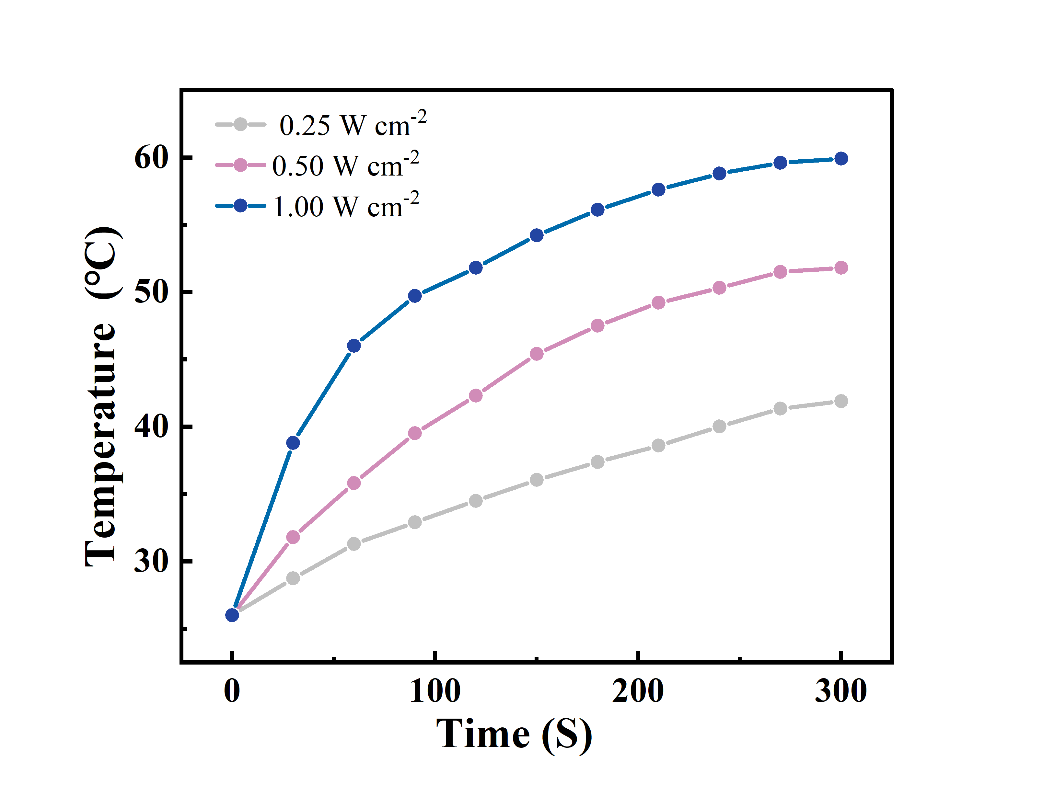


**Figure S8.** Heating curves of APSMR aqueous dispersion under 1064 nm laser illumination at different power densities (0.25, 0.5 and 1.0W cm^-2^) for 5 min.


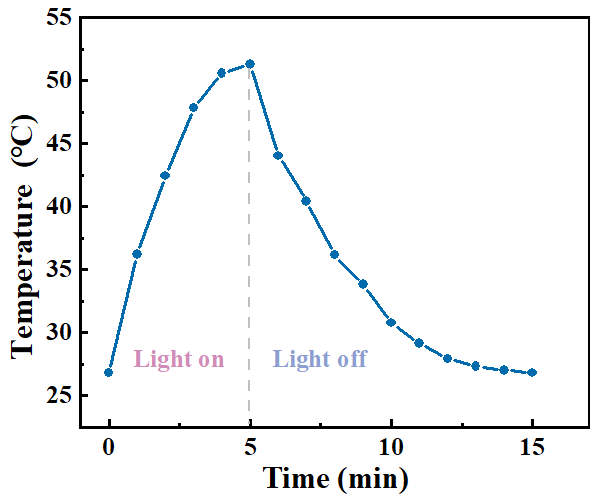


**Figure S9.** Temperature variations in a 100 μg mL^-1^ APSMR aqueous solution influenced by the ON and OFF states of the 1064nm laser.


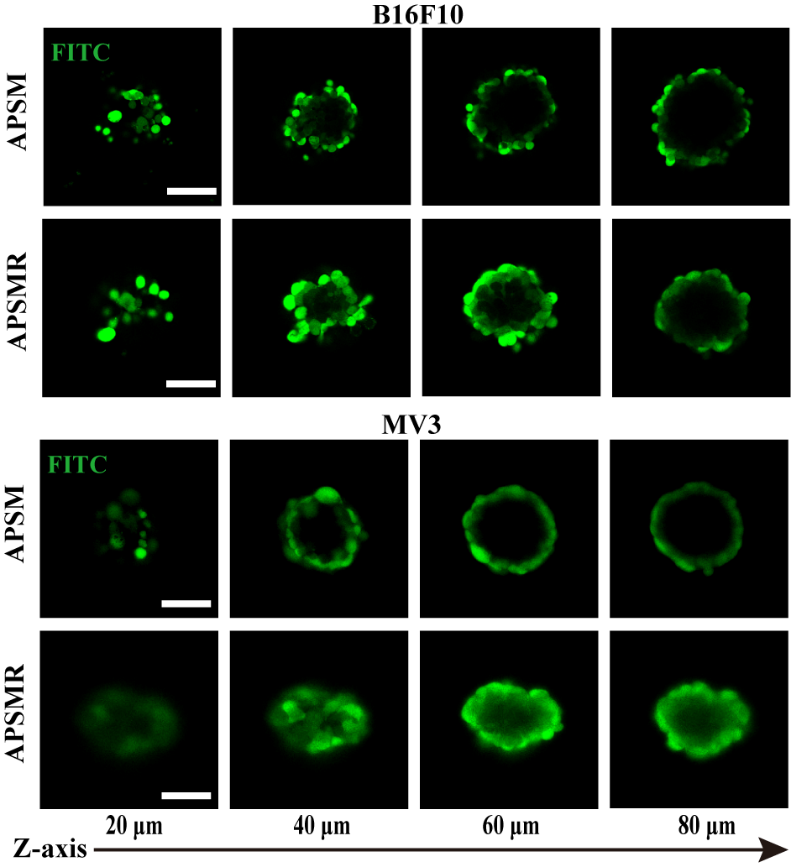


**Figure S10.** CLSM images of B16F10 and MV3 cell spheres uptake of nanoparticles APSM-FITC and APSMR-FITC (scale bar: 100 µm).


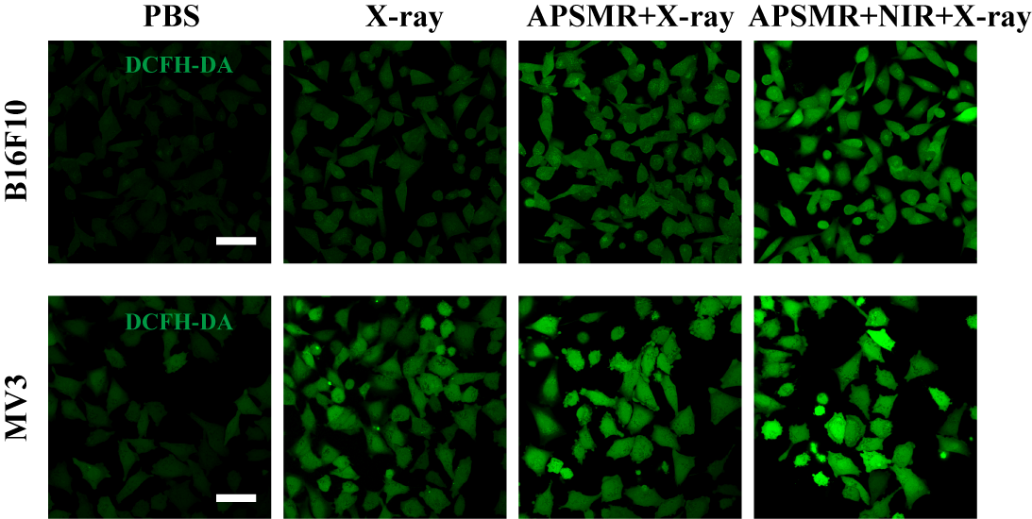


**Figure S11.** CLSM images of intracellular ROS generation in B16F10 and MV3 cells were detected by DCFH-DA after treatment corresponding to grouping (scale bar: 50 µm).


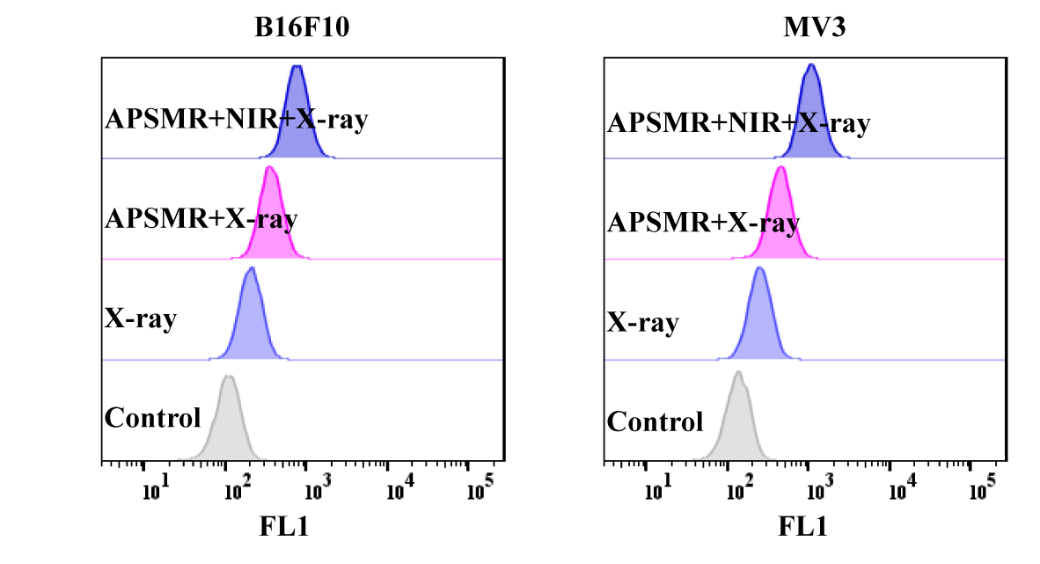


**Figure S12.** Flow analysis of intracellular ROS generation in B16F10 and MV3 cells were detected by DCFH-DA after treatment corresponding to grouping.


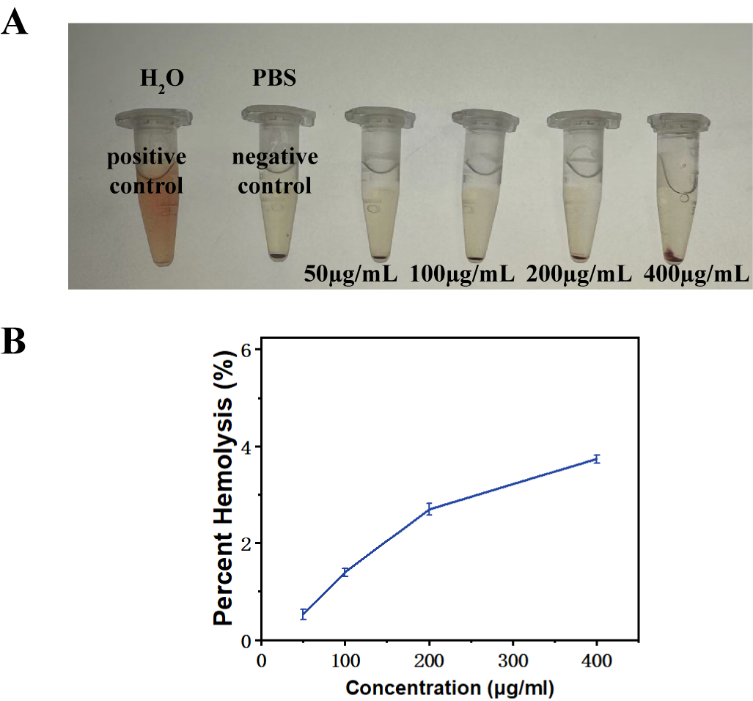


**Figure S13.** (A-B) Hemolysis rate of red blood cells treated with APSMR at increasing concentrations from 50 to 400 µg mL^-1^. PBS is used as a negative control and ddH_2_O is used as a positive control.


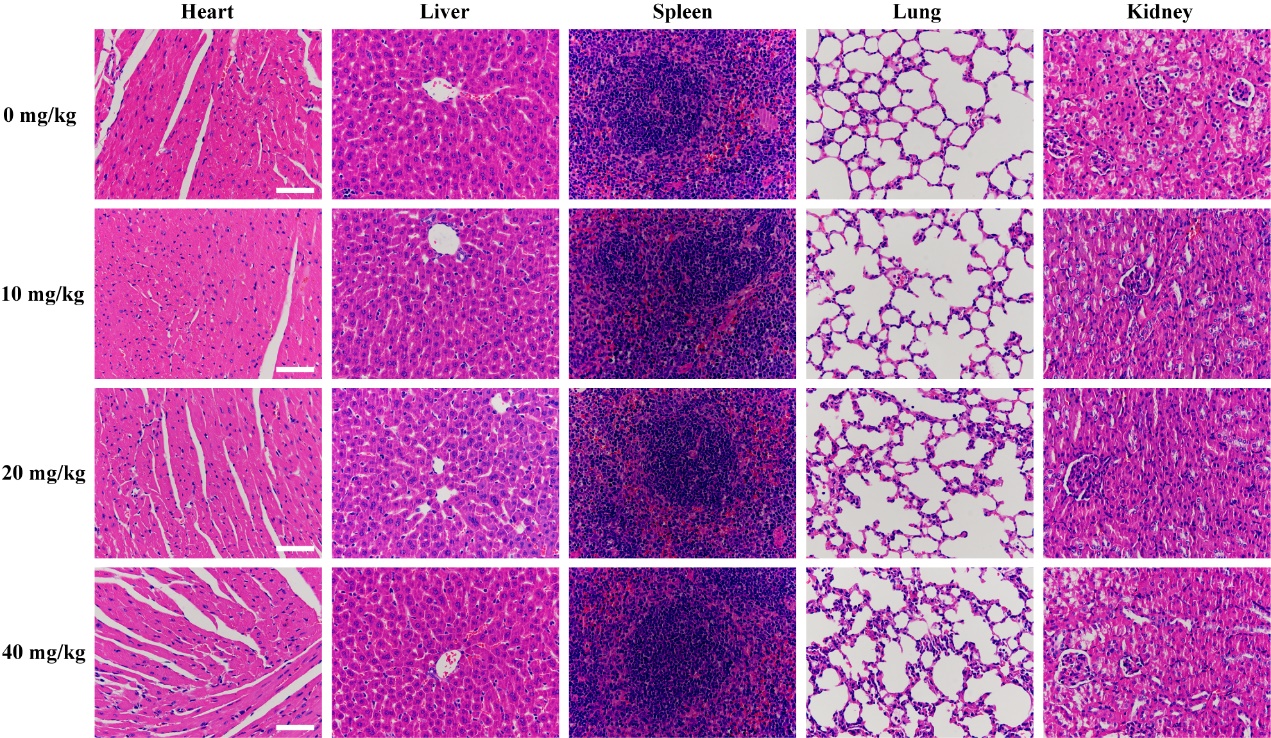


**Figure S14.** H&E staining images of the main organs (heart, liver, spleen, lung, kidney) of mice treated with different APSMR (0mg kg^-1^, 10mg kg^-1^, 20mg kg^-1^, 40mg kg^-1^) concentrations (scale bar: 50 µm).


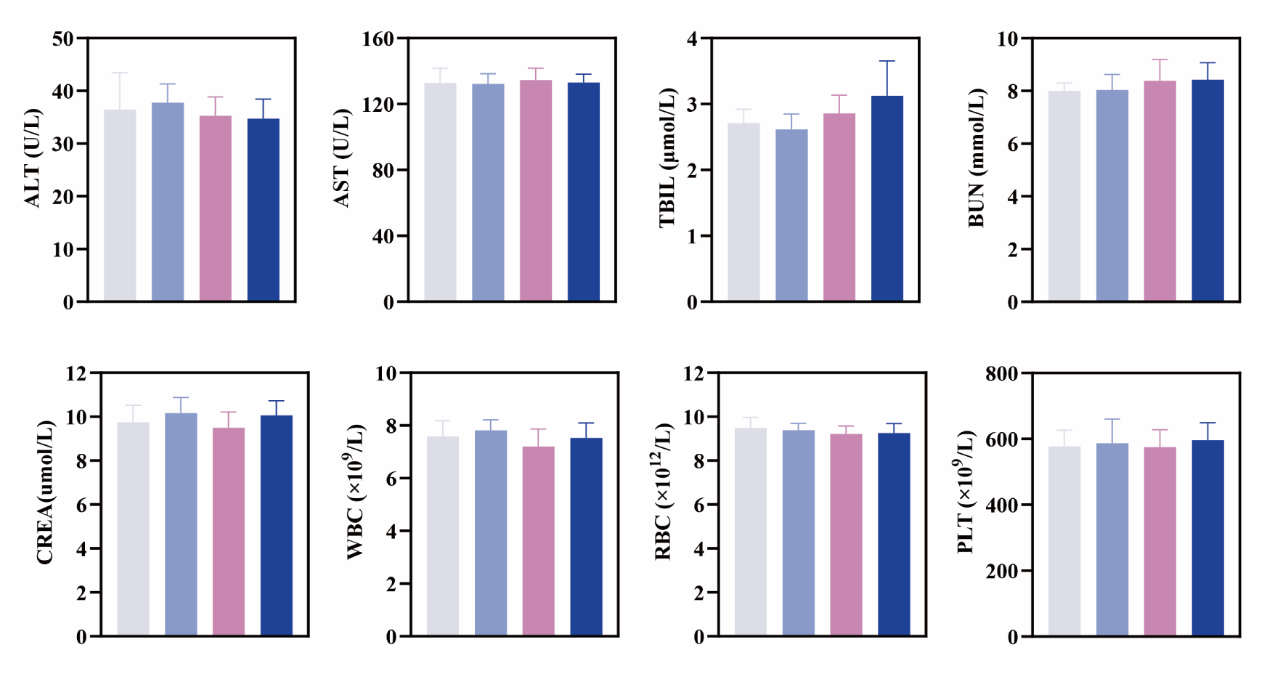


| **Index (Unit)** | **0mg kg^-1^** | **10mg kg^-1^** | **20mg kg^-1^** | **40mg kg^-1^** |
| --- | --- | --- | --- | --- |
| ALT (U/L) | 36.448 | 37.772 | 35.284 | 34.732 |
| AST (U/L) | 132.916 | 132.256 | 134.630 | 133.150 |
| TBIL (μmol/L) | 2.712 | 2.616 | 2.858 | 3.124 |
| BUN (mmol/L) | 8.002 | 8.034 | 8.384 | 8.432 |
| CREA (μmol/L) | 9.746 | 10.168 | 9.496 | 10.068 |
| WBC (10^9^/L) | 7.584 | 7.814 | 7.202 | 7.532 |
| RBC (10^12^/L) | 9.488 | 9.384 | 9.216 | 9.250 |
| PLT (10^9^/ L) | 577.970 | 586.740 | 575.322 | 596.282 |

**Figure S15.** The toxicity of APSMR in mice was evaluated by the changes of blood routine and blood biochemical indexes (AST, ALT, TBIL, BUN, CREA, WBC, RBC, PLT) after treatment with different concentrations of APSMR (0mg kg^-1^, 10mg kg^-1^, 20mg kg^-1^, 40mg kg^-1^).


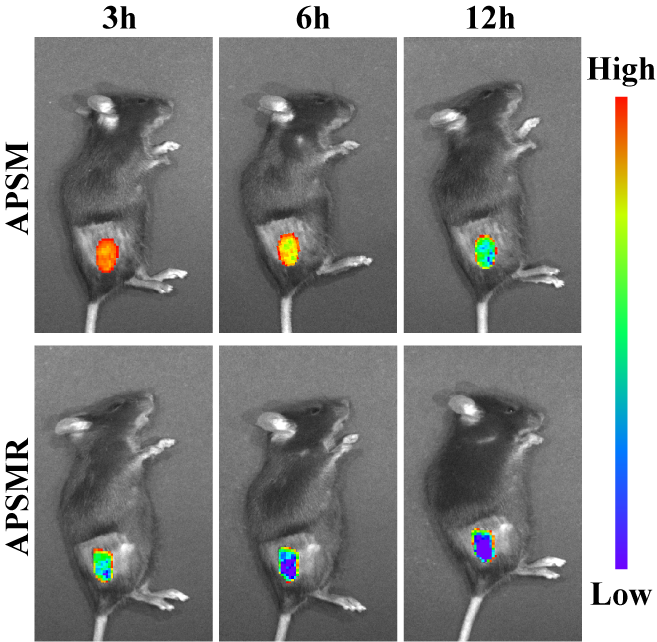


**Figure S16.** Fluorescence images of tumor sites of B16F10 tumor-bearing mice at 3h, 6h, and 12h after intravenous administration of APSM-Cy5 and APSMR-Cy5.


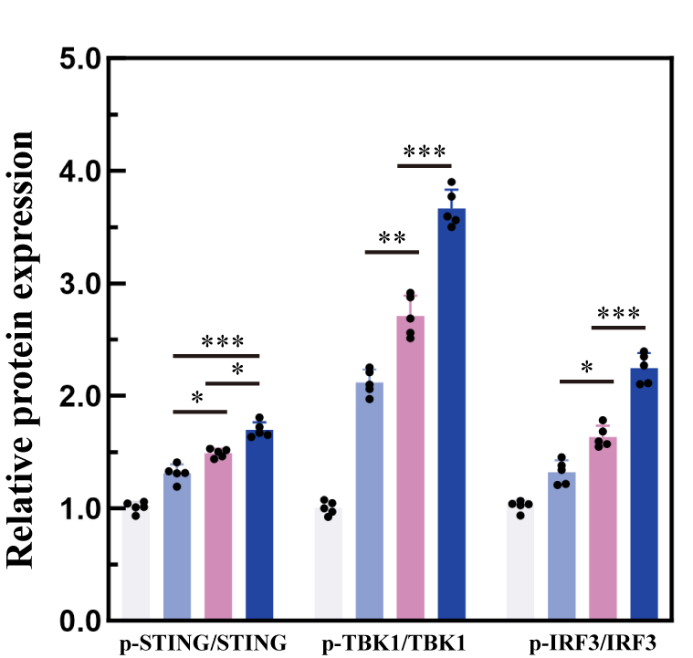


**Figure S17.** Statistical analysis of expression levels of key proteins in cGAS-STING signaling pathway in tumor tissues of different groups after treatment. Data are presented as mean values±SD. **p* < 0.05, ***p* < 0.01, ****p* < 0.001.


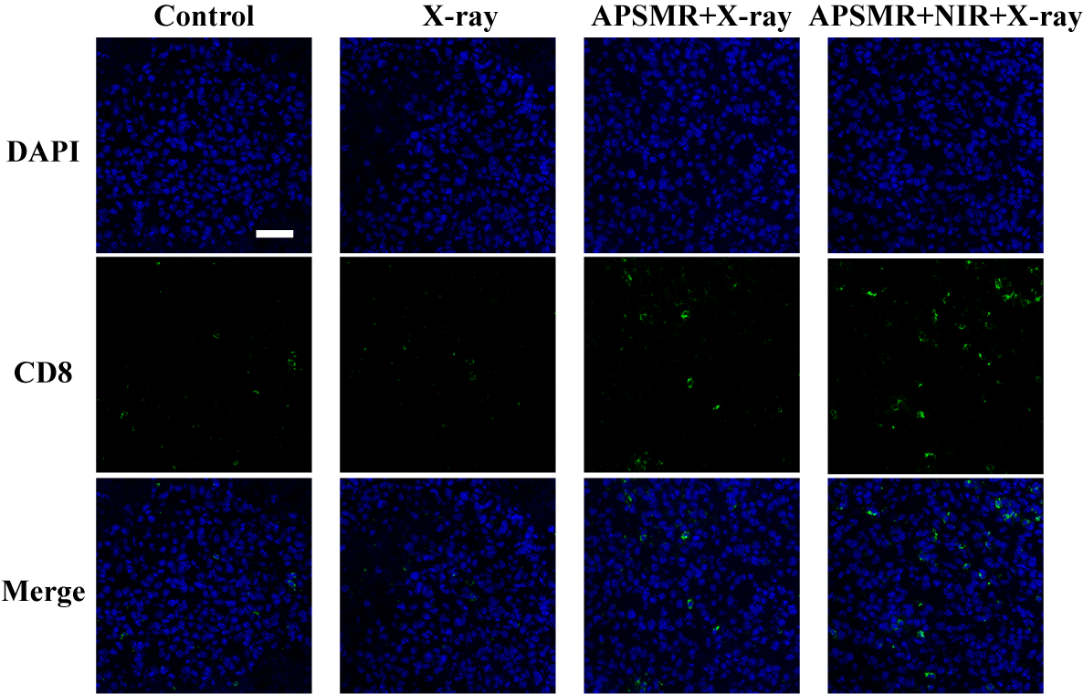


**Figure S18.** Immunofluorescence staining of CD8^+^ T cells in primary tumors tissue (scale bar: 50 μm)


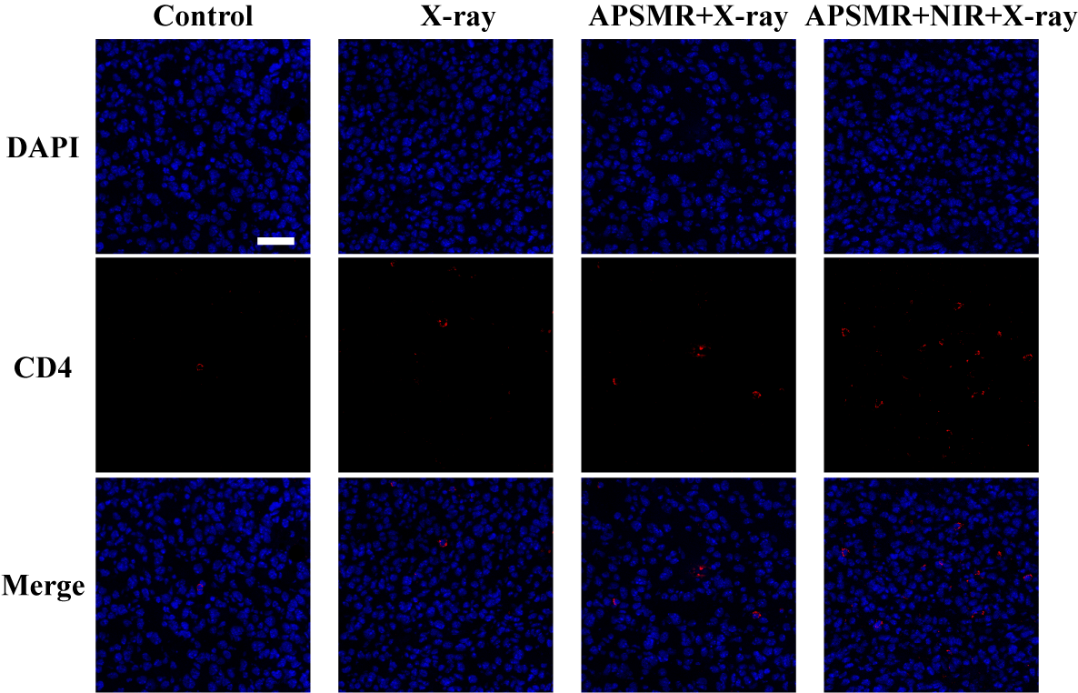


**Figure S19.** Immunofluorescence staining of CD4^+^ T cells in primary tumors tissue (scale bar: 50 μm)


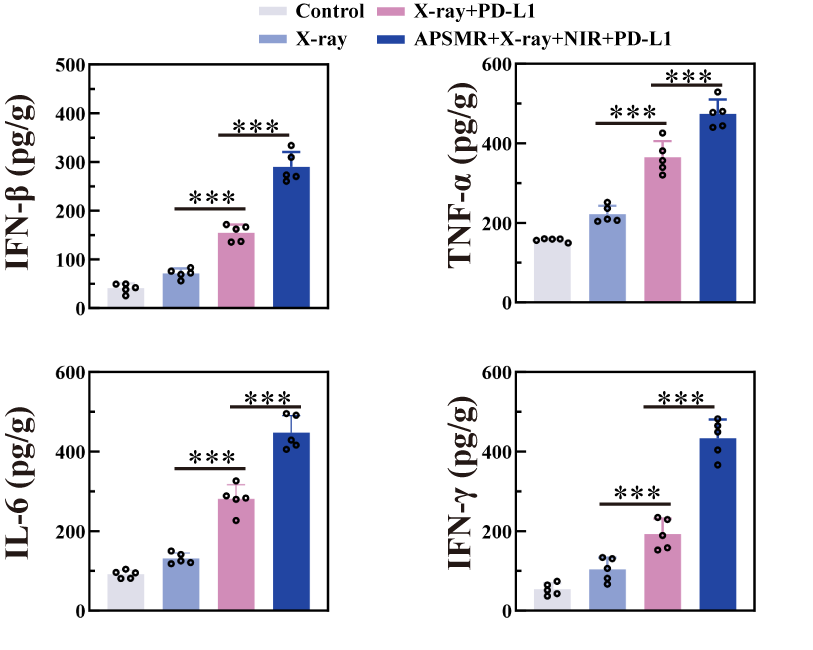


**Figure S20.** ELISA analysis of IFN-β, IL-6, TNF-α, and IFN-γ levels in tumor tissues. Data are presented as mean values±SD. **p* < 0.05, ***p* < 0.01, ****p* < 0.001.
